# Occurrence of virulence determinants in *vibrio cholerae*, *vibrio mimicus*, *vibrio alginolyticus*, and *vibrio parahaemolyticus* isolates from important water resources of Eastern Cape, South Africa

**DOI:** 10.1186/s12866-023-03060-z

**Published:** 2023-10-28

**Authors:** Oluwatayo E. Abioye, Charles A. Osunla, Nolonwabo Nontongana, Anthony I. Okoh

**Affiliations:** 1 Department of Microbiology, Obafemi Awolowo University, Ife, Nigeria; 2 Department of Microbiology, Adekunle Ajasin University, Akungba Akoko, Nigeria; 3 SAMRC Microbial Water Quality Monitoring Centre, University of Fort Hare, Alice, South Africa

**Keywords:** Vibrio species, Virulence genes, Freshwater, Vibriosis, Multiple virulence gene index, Cholera-vibriosis hotspot determination

## Abstract

**Background:**

Virulence determinants are crucial to the risk assessment of pathogens in an environment. This study investigated the presence of eleven key virulence-associated genes in *Vibrio cholerae* (*n =* 111) and *Vibrio mimicus* (*n =* 22) and eight virulence determinants in *Vibrio alginolyticus* (n = 65) and *Vibrio parahaemolyticus* (n = 17) isolated from six important water resources in Eastern Cape, South Africa, using PCR techniques. The multiple virulence gene indexes (MVGI) for sampling sites and isolates as well as hotspots for potential vibriosis outbreaks among sampling sites were determined statistically based on the comparison of MVGI.

**Result:**

The PCR assay showed that all the *V. cholerae* isolates belong to non-O1/non-O139 serogroups. Of the isolates, *Vibrio Cholera* (84%), *V. mimicus* (73%), *V. alginolyticus* (91%) and *V. parahaemolyticus* (100%) isolates harboured at least one of the virulence-associated genes investigated. The virulence gene combinations detected in isolates varied at sampling site and across sites. Typical virulence-associated determinants of *V. cholerae* were detected in *V. mimicus* while that of *V. parahaemolyticus* were detected in *V. alginolyticus*. The isolates with the highest MVGI were recovered from three estuaries (Sunday river, Swartkopps river, buffalo river) and a freshwater resource (Lashinton river). The cumulative MVGI for *V. cholerae*, *V. mimicus*, *V. alginolyticus* and *V. parahaemolyticus* isolates were 0.34, 0.20, 0.45, and 0.40 respectively. The targeted *Vibrio* spp. in increasing order of the public health risk posed in our study areas based on the MVGI is *V. alginolyticus* > *V. parahaemolyticus* > *V. cholerae > V. mimicus*. Five (sites SR, PA5, PA6, EL4 and EL6) out of the seventeen sampling sites were detected as the hotspots for potential cholera-like infection and vibriosis outbreaks.

**Conclusions:**

Our findings suggest that humans having contact with water resources in our study areas are exposed to potential public health risks owing to the detection of virulent determinants in human pathogenic *Vibrio* spp. recovered from the water resources. The study affirms the relevancy of environmental *Vibrio* species to the epidemiology of vibriosis, cholera and cholera-like infections. Hence we suggest a monitoring program for human pathogenic *Vibrio* spp. in the environment most especially surface water that humans have contact with regularly.

**Supplementary Information:**

The online version contains supplementary material available at 10.1186/s12866-023-03060-z.

## Introduction

Water has been identified as one of the earth’s most precious and threatened resources which must be well protected and enhanced for good human health [1]. The causative agents of health challenges that usually emanate from water are diverse nonetheless, the role of bacteria especially the pathogenic ones cannot be overemphasized. *V. cholerae* and its close relative *V. mimicus* are bacteria of public health importance, especially as an etiological agent of waterborne infections. *V. cholerae* serotypes O1 and O139 are famous for the several cholera pandemics recorded over the years while non-O1/non-O139 serotypes have been responsible for several cholera-like outbreaks [2–9]. Also, *V. mimicus* which is a close relative of *V. cholerae* has been implicated in outbreaks of diseases such as gastroenteritis, ear infections and severe cholera-like diarrhoea in the time past [10–12]. The commonly reported virulence determinants of *V. cholera*e irrespective of the serotype are hemolysin (*hlyA*), the actin cross-linking repeats toxin (*rtxA*), hemagglutinin protease (*hap*), type III and VI secretion systems, vibrio pathogenic highland (*vpi*), neuraminidase-encoding (*nanH*) gene, toxin (NAG-ST and ctx genes), accessory cholera enterotoxin (*ace*), *zot* (zonula occludens toxin), transmembrane regulatory protein gene (*toxR*) and toxin-coregulated pili (*tcp*) [2, 9, 13–18]. Other important vibrios pathogenic island (VPI) associated virulence determinants include *ald, tag, toxT, acfB, acfC, orfZ, orfW, int, LJ and RJ* genetic elements. Also, *cep and orfU* are the other two CTX-associated virulence genetic elements of importance that are found in *V. cholerae* [19]. Although it is scarce in the literature, some of the *V. cholerae* and *V. parahaemolyticus* typical genes most especially toxin and toxin regulatory genes have been detected in *V. mimicus* [20].

*Vibrio parahaemolyticus* is an established human pathogen that causes gastroenteritis, wound infections and some other human diseases [21]. The pathogen is the most implicated *Vibrio* spp. in seafood gastroenteritis. The key virulent signatures found in *V. parahaemolyticus* are the thermostable direct hemolysin (TDH), thermor-stable-related hemolysin (TRH) and the thermo labile hemolysin (TLH) genes. Of the three, TDH and TRH genes have been proposed as the most important virulence factors for *V. parahaemolyticus* human infections while the role of TLH gene in *Vibrio parahaemolyticus*related human infections is regarded unclear [21–25]. However, recent studies suggested that thermolabile hemolysin (TLH) gene could be as important as TDH and TRH in human infection episodes [24, 25]. The gene was reported to up-regulate in the human gastrointestinal model and also lyse human erythrocytes [24–27]. *Vibrio alginolyticus* (formerly *V. parahaemolyticus* biotype 2) was considered a non-human pathogen however, it has recently become a bacteria of public health concern because of its involvement in human disease conditions [28]. Although there is a paucity of information on the virulence capability of the organism, the available documentation showed that the organism is a potential reservoir of many virulence genes known in other members of the *Vibrio* genus [29–33]. For example, the *tdh*, *trh* and *tlh* genes and other virulence genes commonly found in *V. parahaemolyticus*, *vopD* gene [30–32]and *vopB* genes belonging to the T3SS, and *vgrG*, *hcp* and *vasH* genes of the T6SS have been detected in *V. alginolyticus* [34]. Aside the waterborne and foodborne infections cause by *V. cholerae* and its close relative *V. mimicus; V. parahaemolyticus* and *V. alginolyticus*, they also cause economic losses in mariculture and aquaculture farms round the globe. Mariculture, aquaculture and recreational fishing are common activities that exposes human to different types of surface water resources in the Eastern Cape Province.

The presence/detection of bacteria in water resources is not enough to ascertain the magnitude of public health risk posed by the bacteria since the degree of pathogenicity, severity and treatability of infections is directly proportional to the number of virulence and resistance genes present/acquired by pathogens [35].

The detection of virulence determinants in the pathogenic bacteria isolated from water resources is essential for microbial risk assessment and this kind of assessment will enhance appropriate decision-making in the epidemiology of pathogenic *Vibrio* species. Hence, we elucidated the presence of virulence determinants in four medically important *Vibrio* species earlier isolated from important water resources in the Eastern Cape as reported in one of our previous studies [36] and determine hotspots for potential cholera and vibriosis outbreaks among our sampling sites.

## Result

The non-detection of *rfb-O1* and *rfb*-O139 genes confirms all *V. cholerae* isolates in this study as members of non-O1/non-O139 serogroup. The prevalence of targeted virulence determinants in isolates from freshwater and brackish water is given in Table 1 while the variability in the gene combinations detected in the *Vibrio* species isolates is given in Table 2. Eighty-four per cent of *V. cholera* (n = 93), 73% of *V. mimicus* (n = 16), 91% of *V. alginolyticus* (n = 60) isolates and 100% of *V. parahaemolytic* (n = 17) harboured at least one of the virulence-associated genes investigated (Tables S1-S8). The gel pictures showing the expected DNA band sizes of the regions of interest on the targeted genes are given in Plates S1-S10.

**Table 1:** Prevalence (%) of targeted virulence genes in isolates from freshwater and brackish water samples

**Organisms/Genes**	***ctxA***		***Zot***		***vpi***	***toxR***		***ompU***		***Tcp***		***ace***		***hyla***	***rtxA***		***rtxC***		***ctxB***	
Vc_Freshwater	AB		AB		27.71	66.27		48.19		8.43		AB		80.72	84.34		84.34		AB	
Vc_Brackishwater	AB		3.45		17.24	51.72		27.59		6.90		AB		58.62	68.97		68.97		AB	
Vm_Freshwater	AB		AB		64.29	28.57		57.14		AB		AB		28.57	50		50		AB	
Vm_Brackishwater	AB		AB		25	12.5		AB		AB		AB		AB	37.5		37.5		AB	
**Organisms/Genes**		***tlh***		***trh***			***tdh***		***vppC***		***vop***		***vgrg***			***hcp***		***vpi***		
Va_Freshwater		20		20			AB		AB		AB		AB			AB		AB		
Va_Brackishwater		88.33		6.67			15.00		61.67		AB		93.33			93.33		26.67		
Vp_Brackishwater		93.75		AB			AB		56.25		6.25		68.75			68.75		43.75		

Key: AB = absent, Vc = *V. cholerae*, Vm = *V. mimicus*, Va = *V. alginolyticus* and Vp = *V. parahaemolyticus*

Note: Results are in percentages

### Variability in virulence genes combination, multiple virulence gene index (MVGI), and determination of hotspots for potential cholera and vibriosis outbreak based on targeted virulence determinants

In this study, different combinations of targeted virulence genes were detected in the *Vibrio* spp. isolates and these are given in Table 2. Nine different gene combinations types were found among *V. cholerae* from brackish water samples while fifteen were found among *V. cholerae* from freshwater samples. Two different combination types were detected among *V. mimicus* from brackish water samples while seven were found among *V. mimicus* recovered from freshwater samples. One combination type was found among *V. alginolyticus* from freshwater but eleven among *V. alginolyticus* from brackish water samples. Seven different virulence gene combination types were found among *V. parahaemolyticus* isolates from brackish water samples. The site with the most diverse *Vibrio* species base on virulence gene combination types detected was SKR. The list of isolates with their corresponding sampling site and characteristic MVGI is given in Tables S1-S8. The CMVGI for the isolates and sampling sites is given in Table 3 with *V. cholerae* having the highest CMVGI of 0.36 among isolates from fresh water. On the other hand, *V. alginolyticus* had the highest CMVGI of 0.48 among isolates from brackish water. Site EL4 has the highest CMVGI of 0.45 among freshwater sampling sites while site SR has the highest CMVGI of 0.54 among the sites from brackish water sampling sites. Leven test revealed that only MVGI data for freshwater isolates is parametric variable while MVGI data for brackish water isolates, freshwater and brackish water sampling sites are non-parametric variables. The ANOVA showed that mean CMVGI for freshwater isolates are significantly different while Welch ANOVA showed that mean CMVGI for brackish water isolates, freshwater and brackish water sampling sites are significantly different. The benferroni Post Hoc test for fresh water isolates showed that CMVGI for *V. cholerae* and *V. mimicus* are not significantly different while Game-Howell Post Hoc test showed that CMVGI of *V. alginolytiucs* and *V. parahaemolyticus* of the isolates from brackish water were not significantly differenttoo. Hence, of the four *Vibrio* spp. used for this study, *V. cholerae* and *V. mimicus* are the most probable organisms to cause vibrio-related infections at the freshwater sampling environ while *V.* alginolytiucs and *V. parahaemolyticus* are most likely bacteria to cause vibrio-related infections at the brackish water sampling sites based on criterion set in section 2.3. Of the brackish water sampling sites, the Game-Howell Post Hoc test revealed that site SR’s CMVGI is significantly higher than that of other four brackish water sampling sites (PA7, SKR, EL5 and EL6). However, the CMVGI of those four other brackish water sampling sites despite been numerically different in the order EL6 > SKR > EL5 > PA7, are not statistically different. On the other hand, sites PA1, PA2, PA3, PA5 and PA6 have CMVGI that are not significantly different from CMVGI of site EL4 which has the highest CMVGI among the nine freshwater sampling sites. Therefore, the statistical result suggested sites PA1, PA2, PA3, PA5, PA6 and EL4 as freshwater hotspots and site SR as the brackish water hotspot for possible relatively high vibrio-related infections outbreaks among the sixteen sampling sites from which isolates used for this study were recovered. The ANOVA and Welch statistical test results are given in Table 4 while Post Hoc test results are in Table S10.

**Table 2:** Different virulence gene combinations detected in isolates

Isolates	Water Types	Virulence gene combination detected	n	Sampling site (number of isolates)
*Vibrio cholerae*	Brackish Water	hyla,rtxA,rtxC	3	SKR (2), PA7 (1)
		ompU,hylA,rtxA,rtxC	2	SKR
		toxR,hylA,rtxA,rtxC	5	PA7 (3), SKR (2)
		toxR,ompU,hylA,rtxA,rtxC	2	SKR
		toxR,ompU,rtxA,rtxC	1	SKR
		toxR,rtxA,rtxC	2	EL5
		vpi,toxR,ompU,hylA,rtxA,rtxC	2	SKR
		vpi,toxR,tcp,hylA,rtxA,rtxC	2	SR
		Zot,vpi,toxR,ompU,hylA,rtxA,rtxC	1	SR
	Freshwater	hylA	1	EL3
		hylA,rtxA,rtxC	5	PA6
		ompU,hylA,rtxA,rtxC	5	PA5 (2), PA6 (3)
		ompU,rtxA,rtxC	1	PA4(1)
		toxR	3	PA2 (1), PA3 (2)
		toxR,hylA,rtxA,rtxC	8	PA5 (6), PA6 (2)
		toxR,ompU,hylA,rtxA,rtxC	27	EL4 (3), PA1 (1), PA3 (1), PA4 (2), PA5 (5), PA6 (15)
		toxR,rtxA,rtxC	2	PA5
		vpi,hylA,rtxA,rtxC	2	PA2 (1), PA4 (1)
		vpi,ompU,hylA,rtxA,rtxC	1	PA6
		vpi,rtxA,rtxC	1	PA4
		vpi,tcp,hylA,rtxA,rtxC	2	PA5
		vpi,toxR,hylA,rtxA,rtxC	7	PA5 (2), PA6 (5)
		vpi,toxR,ompU,hylA,rtxA,rtxC	4	PA2 (2), PA4 (1), PA6 (1)
		vpi,toxR,ompU,tcp,hylA,rtxA,rtxC	1	PA5
		vpi,toxR,tcp,hylA,rtxA,rtxC	4	PA5
*Vibrio mimicus*	Brackish Water	vpi,rtxA,rtxC	2	EL5 (1), EL6 (1)
		toxR,rtxA,rtxC	1	EL6 (1)
	Freshwater	ompU	2	EL2
		ToxR	1	PA3
		vpi,hylA,rtxA,rtxC	2	PA4
		vpi,ompU	2	PA2 (1), EL2 (1)
		vpi,ompU,rtxA,rtxC	3	PA3 (1), PA5 (2)
		vpi,toxR	1	EL1
		vpi,toxR,hylA,rtxA,rtxC	2	PA4
*Vibrio alginolyticus*	Brackish	tdh,vgrg,hcp	1	EL6
		tlh,tdh,trh	1	SKR
		tlh,tdh,vpc,vgrg,hcp	6	EL5(4),EL6(1),SKR(1)
		tlh,trh,vgrg,hcp	2	EL6
		tlh,vgrg,hcp	11	EL5(3), EL6(7),PA7(1)
		tlh,vgrg,hcp,vpi	5	EL6
		tlh,vpc	1	SKR
		tlh,vpc,vgrg,hcp	16	PA7 (1), SR (8), SKR (7)
		tlh,vpc,vgrg,hcp,vpi	10	SKR (1), SR (10)
		vgrg,hcp	2	EL6
		vpc,vgrg,hcp	2	SKR
	Freshwater	tlh,trh	1	PA4
*Vibrio parahaemolyticus*	Brackish	tlh	2	SKR
		tlh,vgrg,hcp	1	EL5
		tlh,vgrg,hcp,vpi	3	EL6 (2),PA7 (1)
		tlh,vpc,vgrg,hcp	5	SKR (1), SR (4)
		tlh,vpc,vgrg,hcp,vpi	1	SKR
		tlh,vpc,vpi	3	SKR
		vop,vgrg,hcp	1	PA7
	Freshwater	tdh	1	ALD2

Keys: ND = None of the targeted virulence determinants detected in the isolate, n = number of isolates

**Table 3:** Descriptive statistics of Multiple virulence gene indexes (MVGI) of isolates and sampling sites

MVGI of isolates from Freshwater								
	N	Mean	Std. Deviation	Std. Error	95% Confidence Interval for Mean		Minimum	Maximum
					Lower Bound	Upper Bound		
VC	82	0.3581	0.16246	0.01794	0.3224	0.3938	0.00	0.55
VM	14	0.2532	0.15203	0.04063	0.1655	0.3410	0.00	0.45
VA	5	0.0250	0.05590	0.02500	− 0.0444	0.0944	0.00	0.13
Total	101	0.3271	0.17500	0.01741	0.2925	0.3616	0.00	0.55
MVGI of isolates from Brackish water								
	N	Mean	Std. Deviation	Std. Error	95% Confidence Interval for Mean		Minimum	Maximum
					Lower Bound	Upper Bound		
VC	29	0.2759	0.20969	0.03894	0.1961	0.3556	0.00	0.64
VM	8	0.1023	0.14115	0.04990	− 0.0157	0.2203	0.00	0.27
VA	60	0.4750	0.13961	0.01802	0.4389	0.5111	0.00	0.63
VP	16	0.4219	0.13598	0.03399	0.3494	0.4943	0.13	0.63
Total	113	0.3900	0.19566	0.01841	0.3535	0.4265	0.00	0.64
MVGI freshwater sampling sites								
	N	Mean	Std. Deviation	Std. Error	95% Confidence Interval for Mean		Minimum	Maximum
					Lower Bound	Upper Bound		
PA1	2	0.2273	0.32141	0.22727	-2.6605	3.1150	0.00	0.45
PA2	7	0.2468	0.23888	0.09029	0.0258	0.4677	0.00	0.55
PA3	6	0.1818	0.18182	0.07423	− 0.0090	0.3726	0.00	0.45
PA4	16	0.2464	0.20771	0.05193	0.1358	0.3571	0.00	0.55
PA5	26	0.4056	0.10035	0.01968	0.3651	0.4461	0.09	0.55
PA6	33	0.4022	0.10172	0.01771	0.3661	0.4383	0.00	0.55
EL1	2	0.1818	0.00000	0.00000	0.1818	0.1818	0.18	0.18
EL2	2	0.0909	0.00000	0.00000	0.0909	0.0909	0.09	0.09
EL4	3	0.4545	0.00000	0.00000	0.4545	0.4545	0.45	0.45
ALD2	4	0.0313	0.06250	0.03125	− 0.0682	0.1307	0.00	0.13
Total	101	0.3274	0.17457	0.01737	0.2929	0.3619	0.00	0.55
MVGI brackish water sampling sites								
	N	Mean	Std. Deviation	Std. Error	95% Confidence Interval for Mean		Minimum	Maximum
					Lower Bound	Upper Bound		
PA7	11	0.2831	0.19261	0.05807	0.1537	0.4125	0.00	0.50
EL5	17	0.2834	0.24781	0.06010	0.1560	0.4108	0.00	0.63
EL6	23	0.3987	0.13194	0.02751	0.3417	0.4558	0.00	0.63
SR	26	0.5376	0.12526	0.02457	0.4870	0.5882	0.00	0.64
SKR	36	0.3608	0.18832	0.03139	0.2971	0.4245	0.00	0.63
Total	113	0.3900	0.19566	0.01841	0.3535	0.4265	0.00	0.64

Key: VC = *V. cholerae*, VM = *V. mimicus*, VA = *V. alginolyticus*, VP = *V. parahaemolyticus*, MVGI = multiple virulence gene index

**Table 4:** Statistical comparison of the CMVGI of four *Vibrio* spp. Isolates and the sampling sites

CMVGI ANOVA for VC, VM and VA from freshwater					
	Sum of Squares	df	Mean Square	F	Sig.
Between Groups	0.611	2	0.306	12.224	< 0.001
Within Groups	2.451	98	0.025		
Total	3.062	100			
CMVGI ANOVA for VC, VM and VA from brackish water					
	Statistic	df1	df2	Sig.	
Welch	19.963	3	25.524	< 0.001	
Brown-Forsythe	19.549	3	55.145	< 0.001	
CMVGI ANOVA for brackish water sampling sites					
	Statistic	df1	df2	Sig.	
Welch	8.959	4	40.188	< 0.001	
Brown-Forsythe	6.759	4	58.796	< 0.001	
CMVGI ANOVA for freshwater sampling sites					
	Statistic	df1	df2	Sig.	
Welch	3460.335	9	12.845	< 0.001	
Brown-Forsythe	5.917	9	5.26	0.028	

Key: VC = *V. cholerae*, VM = *V. mimicus*, VA = *V. alginolyticus*, VP = *V. parahaemolyticus*,C MVGI = cumulative multiple virulence gene index

## Discussion

This study reveals the prevalence of eleven virulence genes in the non-O1/non-O139 *V. cholerae* and *V. mimicus* and eight virulence genes in *V. alginolyticus* and *V. parahaemolyticus* isolates. The low prevalence of key cholera-associated virulence factors (*zot, tcp, ctx* and *ace*) and the relatively high prevalence of other virulence-associated factors (*hyla, rtx, toxR*, *ompU*, vpi) in the non-O1/non-O139 *V. cholerae* is in concordance with the previous studies [9, 18, 37–45]. The two most essential virulence genes for cholera epidemics and pandemics are *tcp* and *ctx* genes [46–48]. Interestingly, some earlier reports detected the two genes in non-O1/non-O139 *V. cholerae* strains [49, 50] such as O141, O75, O27, O37, O53, and O65 strains [51–55] while some others like the current study did not [56–59]. The presence of the two genes in non-O1/non-O139 strains suggests that toxigenic non-O1/non-O139 strains of *V. cholerae* exist. Although CTX^+^ and TCP^+^*V. cholerae* non-O1/non-O139 were not encountered in the present study, *ctx*^−^ but *tcp*^+^*V. cholerae* non-O1/non-O139 was detected and the ability of *ctx*^−^and *tcp*^−^ to acquire the two genes has been reported [60, 61]. Although *Vibrio cholerae* non-O1/non-O139 strains even those carrying *tcp* and *ctx* do not cause cholera epidemic/pandemic, they have been implicated in vibrioses such as gastroenteritis, ear infections, septicemia, and cholera-like infections which are sometimes severe and fatal most especially in immunocompromised patients [8, 59, 62, 63]. The work of [64] showed that several other virulence determinants like those found in *V. cholerae* in this study work in synergy and are responsible for the pathogenicity of non-O1/non-O139 serogroups of *V. cholerae* that are negative for *tcp* and *ctx* genes. The non-detection of the *ctx* gene and detection of a relatively low *tcp*^+^*V cholerae* in this study does not guarantee that our sampling areas are free of possible future cholera or fatal vibrio related diarrheal disease outbreaks for three reasons. Firstly, molecular studies have shown that the non-toxigenic strain of *V. cholerae* can be transformed into toxigenic strain by CTXφ (*V. cholerae* phage) infestation and antigenic shift that results from the homologous recombination-mediated exchange of O-antigen biosynthesis (wb*) clusters between toxigenic and non-toxigenic strains of *V. cholerae* [55, 65]. Secondly, the isolation of *tcp*^+^ but ctx^−^*V. cholerae* strains from Sunday and Kowie River water samples suggests that the two rivers are potential hotspots for cholera causing *Vibrio cholerae* in the future since *tcp* gene plays a pivotal role in *V. cholerae* pathogenicity in term of the cholera outbreak. The toxin coregulated pili (TCP) gene detected in *V. cholerae* from the two rivers acts as a receptor for CTXφ and when CTXφ infests non-toxigenic *V. cholerae*, it can lead to the emergence of a new toxigenic strain [61, 66]. Thirdly, previous works have shown that Type III Secretion system (T3SS) is relatively common in non-O1/non-O139 *V. cholerae*, and the T3SS^+^ non-O1/non-O139 *V. cholerae* causes severe and fatal diarrhea even more rapidly than *V. cholerae* O1 in animals model. The T3SS is not commonly found in *V. cholerae* O1 and O139 serogroups [18, 67–70]. Also, *toxR*, *vpi, ompU, hyla, and rtx* virulence genes are important drivers of *Vibrio* spp. related infections. The relatively high prevalence of the five aforementioned genes in *Vibrio* spp. isolates in this study suggests that a sizable number of the *V. cholerae* and *V. mimicus* isolates in our study area have virulence gene architecture that only need to acquire a few more virulence determinants to become epidemics causing *Vibrio* species. The high prevalence of *ToxR* gene in *V. cholerae* and *V. mimicus* isolates further affirms the clinical importance of the isolates. *Vibrio* spp. with this gene will possibly not find it difficult to express their virulence determinants in a suitable host such as humans since the virulence functions of *V. cholerae* and *V. mimicus* are regulated by the transmembrane protein encoded by *ToxR* gene. [34, 71]. The VPI region of *Vibrio* genome usually has many features that are typical of pathogenicity islands and these include presence of low G + C content (35%) compared to the rest of the genome (48%), phage-like attachment (*att*), a transposase-like gene, and a phage-like integrase gene (*int*) among others [72]. The *ompU* gene is essential for the adhesion of *Vibrio* spp. to its host and reduces the permeation of antibiotics across the membrane barriers [73]. The *hylA* gene is an important virulence determinant of non-O1 and non-O139 *Vibrio cholerae*. It encodes the hemolysin gene, which plays a very important role in cytotoxicity and apoptosis [74].

*Vibrio mimicus* had been implicated in both waterborne and foodborne disease outbreaks [10, 75, 76] however, there is paucity of information on the virulence determinants of *V. mimicus* in the literature. *Vibrio mimicus* causes infections such as gastroenteritis, ear infections, and severe cholera-like diarrhea. Some virulence determinants peculiar to *V. cholerae* have been detected in *V. mimicus* isolates [11, 77]. In this study, all the virulence-associated genes detected in *V. cholerae* were also detected in at least one *V. mimicus* isolate except for *zot* and *tcp* genes. This finding supports some of the few reports that have shown that *V. mimicus* could carry similar virulence determinants cassette commonly found in *V. cholerae* [10, 20, 78] and this could have something to do with both species sharing common ancestors [77]. The presence of typical *V. cholerae* virulence determinants in *Vibrio mimicus* suggests that the bacterium should be one of the *Vibrio* pathogens to be investigated during cholera outbreaks especially when *Vibrio cholerae* cannot be isolated from samples collected for investigation.

The relatively high prevalence of typical *Vibrio parahaemolyticus* virulence genes in *Vibrio alginolyticus* in this study supports earlier reports that showed that most *Vibrio* spp. possesses atypical virulence genes in addition to their typical virulence determinants [79]. For example, the work of [80] detected *V. cholerae ctxAB* gene in *V. diabolicu, V. alginolyticus* and *V. parahaemolyticus* while studies carried out by [30] and [81] reported *V. parahaemolyticus tdh* gene in *V. harveyi* and *V. mimicus* respectively. Also, *V. cholerae* virulence determinants specifically *zot*, *ace* and *vpi* genes, have been detected in *V. alginolyticus* [32, 33]. *V. parahaemolyticus* is one of the important indicators recommended for accessing the microbial quality of seafood but *V. alginolyticus* is not [82]. The findings of this study and that of earlier reports show that it is important to investigate both *V. alginolyticus* and *V. parahaemolyticus* simultaneously whenever vibriosis peculiar to *V. parahaemolyticus* is suspected or in the microbial assessment of seafood that requires testing for *V. parahaemolyticus*.

In this study, none of the targeted virulence genes was detected in some of our isolates Tables S1-S8. However, these isolates are potential agents of vibrio-related infections since they can acquire virulence factors via horizontal gene transfer. Horizontal gene transfer and recombination are common phenomena that play significant roles in the evolution and emergence of new pathogenic strains among Gram-negative organisms [83, 84]. It is common knowledge that horizontal gene transfer plays a vital role in the exchange of genetic materials among *Vibrio* spp. [84–89].

It was observed in this study that the four targeted *Vibrio* species with varying combinations of virulence determinants co-existed in the same ecological niche and this has been reported in some earlier work [9, 29, 37, 79, 80, 90, 91]. The most diverse *Vibrio* spp. in this study based on the different combinations of the targeted virulence determinants were found at sampling sites PA4, PA5, PA6, PA7, EL6, SR, and SKR where the level of pollution and anthropogenic activities were relatively high. Pollution promotes horizontal gene transfer and enhances bacterial virulence and antibiotic resistance [79, 92]. This could explain the high variability in terms of virulence determinants combinations found among population of each of the four targeted *Vibrio* spp. at the sampling sites PA4, PA5, PA6, PA7, EL6, SR, and SKR. The presence of strains with relatively high variability in virulence-associated gene combinations found at these seven sampling sites is of great public health concern. It has been shown that each virulence determinant that makes up the virulence determinant combinations found in the strains interact synergistically to achieve disease conditions [64]. The presence of different strains of *V. cholerae*, *V. mimicus*, *V. alginolyticus* and *V. parahaemolyticus* harboring various virulence determinants combinations in the water resources sampled interfere with the resourcefulness of the water resources. Water resources contaminated with these pathogens are not good for drinking, irrigation, recreational and agricultural purposes because it has been established that such water resources could lead to disease outbreaks [8, 93, 94]. As such, the presence of virulence determinants carrying *Vibrio* spp. in the water resources especially the freshwater resources in a province that suffers yearly water scarcity [95–97] calls for concern since surface water are key and integral part of water supply in South Africa. People most especially in the rural areas in the absence of sustainable access to potable water, seek for alternative sources to meet their fundamental needs, and surface water is the first point of call as it is easy to access and use [98–101].

There was no known cholera outbreak ongoing when the isolates that were used for this study were recovered from the aquatic milieu and this we believe, accounted for our inability to detect the cholera pandemic and epidemic-causing *V. cholerae* O1 and O139 serotypes among the *V. cholerae* isolates in our study. Nevertheless, the possibility of future outbreaks of vibrio-related illnesses including cholera has been articulated above. Also, the current virulence signature of the *V. cholerae* and *V. mimicus* suggests that some of the sampling areas where the isolates were recovered are at the risk of vibriosis and immunocompromised individuals such as people with HIV and tuberculosis are at higher risk [102]. Compared to other provinces in South Africa, the burden of HIV and tuberculosis is relatively high in Eastern Cape Province most especially in the Sarah Baartman district municipality [94, 103, 104]. Unfortunately, 80%, 40%, 76%, and 63% of the virulence containing *V. cholerae* non-O1/non-O139, *V. mimicus*, *V. alginolyticus* and *V. parahaemolyticus* respectively were recovered from water resources in this district municipality.

In summary, this study which is the first of its kind in the study area shows that the four *Vibrio* species isolates investigated especially those from water resources in Sarah Baartman district municipality, have the potential to cause cholera-like infection and other vibrioses. The virulence gene combinations detected in isolates varied at each sampling site and across sites. All the water resources that harbored the various genotypes (based on the virulence genes combinations) of the four *Vibrio* spp. are important water resources that are normally used for agricultural (fishing and irrigation), recreational and spiritual ablution purposes.

Furthermore, typical virulence-associated determinants of *V. cholerae* (*vpi, toxR, ompU, tcp, hyla, rtxA and rtxC*) were detected in *V. mimicus* while that of *V. parahaemolyticus* (*vpi*, *tlh*, *vppC*, *vopB2*, *vgrG* and *hcp*) were detected in *V. alginoyiticus*. The isolates with the highest MVGI among each of the four targeted *Vibrio* spp. were recovered from three estuaries (Sunday river, Swartkopps river, buffalo river) and a freshwater resource (Lashinton river). The cumulative MVGI for *V. cholerae*, *V. mimicus*, *V. alginolyticus* and *V. parahaemolyticus* isolates were 0.34, 0.20, 0.45, and 0.40 respectively. The presence of pathogenic *Vibrio* spp. in the water resources especially the freshwater resources in a country that is freshwater stressed, calls for concern. The targeted pathogens in increasing order of public health risk posed based on the MVGI can be represented as *V. alginolyticus* > *V.* parahaemolyticus > *V. cholerae > V. mimicus*. Eight (sites SR, PA1, PA2, PA3, PA5, PA6, EL4, and EL6) out of the sixteen sampling sites were detected as the hotspots for potential cholera and vibriosis based on MVGI analysis. Although the MVGI analysis for brackish water sampling sites detected only site SR as the hotspot for vibrio-related infections among the brackish water sampling sites, sites EL6 and SKR can also be considered as important hotspot for vibrio-related infections since they have CMVGI of approximately 0.4. The virulence genes detected in the four medically important *Vibrio* spp. isolates in this study are key to the pathophysiology of cholera and vibriosis. Therefore, to prevent outbreaks of vibrio-related infections in the study area, epidemiological proactive measures such as creation of awareness and Vibrio monitoring programes for our sampling sites most especially rivers where the hotspots are located (Kowie, Bloukrans, Lashinton, Kubusi, Buffallo, Swartkopps and Sunday rivers) is hereby advocated. The relatively high prevalence of virulence-associated genes in isolates from water resources where the level of pollution was relatively high suggests the need to prioritize and enforce hygiene as a major component of the monitoring program.

## Contribution to knowledge

The information on the virulence capability of vibrio isolates from the aquatic environment in Eastern Cape Province is limited in the literature. We believe that the data generated from our study are relevant to microbial risk assessment of *Vibrio* spp. and support the development of environmental regulations that will help in preventing cholera and vibriosis outbreaks at our sampling sites most especially the hotspots. The study also provides information on the virulence of *V. mimicus* and *V. alginolyticus*, two *Vibrio* spp. that are not commonly reported from water sources.

## Materials and methods

### Isolates

The *V. cholerae* (*n =* 111), *V. mimicus* (*n* = 22), *V. alginolyticus* (*n =* 65) and *V. parahaemolyticus* (n = 17) used for this study were environmental isolates recovered from water samples collected from six important water resources in Eastern Cape, Province, South Africa as earlier reported [36]. The water resources from which the isolates were recovered include Kowie River and two of its tributaries, Sunday River, Swartkopps River, Buffalo River, Kubusi River and one of its tributaries, and two of the University of Fort Hare’s Dams.

### DNA extraction and PCR assay

An 18 h old culture of all isolates was prepared from 20% glycerol stock culture stored at -80 ^o^C using freshly prepared sterile brain heart infusion broth. The isolates were afterwards streaked out on a freshly prepared nutrient agar plate containing 1% NaCl and incubated at 37 ^o^C for 18 h. After the incubation period, using the boiling method, the genetic material of a distinct colony of isolates was extracted as described by [36] and used as the template for the PCR assay. Firstly, *V. cholerae* isolates were delineated into O1, O139 or non-O1/non-O139 strains using *rfb-O1* primer specific for *V. cholerae* O1 strain and *rfb*-O139 primer specific for O139 strain. This was followed by the molecular detection of eleven virulence determinants (*rtxA*, *rtxC*, *toxR*, *ompU*, *ctx*, *ctxB*, *vpi*, *hylA*, *tcpA*, *Zot*, *ace*) in *V. cholerae* and *V. mimicus* isolates and eighth virulence determinants (*vpi*, *tdh*, *trh*, *tlh*, *vppC*, *vopB2*, *vgrG*, *hcp*) in *V. alginolyticus* and *V. parahaemolyticus*. The primers sequences, expected amplicon sizes and thermal cycler conditions employed in this study and the PCR type employed for the amplification of the specific regions of the targeted genes are given in the Table S9. The duplex and singleplex PCR assays were carried out in a 25 µl reaction mixture. The reaction mixture and volume of each component of the mixture are as enunciated in an earlier study [105]. Amplicons sizes were resolved on 1.5% agarose gel and the resulting gel was stained with ethidium bromide (1 µg/mL) and viewed under a transilluminator. A reaction mixture without the DNA template and *E. coli* ATCC 35,150 was used as negative and internal controls for the PCR assays respectively.

### Multiple virulence gene index (MVGI) determination and statistical analysis

The multiple virulence gene indexes (MVGI) were determined using the equation I below as earlier reported [79]. The cumulative multiple virulence gene indexes (CMVGI) for the freshwater, brackish water sampling sites, and each of the four *Vibrio* spp. that were targeted in this study were determined using equation II below.

I $$MVGI{\text{ }} = {\text{ }}VGD/VGT$$

II $$CMVGI{\text{ }} = {\text{ }}AVGD/\left( {VGT{\text{ }}x{\text{ }}NI} \right)$$

Where MVGI = multiple virulence gene index, CMVGI = cumulative multiple virulence gene index, VGD = total number of virulence genes detected, VGT = total number of virulence genes targeted, AVGD = aggregate virulence gene detected, NI = number of isolates.

The presence or absence of pollution at the water sampling sites was determined using criteria (color, turbidity, temperature, suspended solids, foam) for determining the presence of physical pollutants [106]. To understand the association between pollution and the prevalence of virulence genes in isolates, a correlation analysis was carried out on the MVGI of isolates versus the detection of pollution at the sampling sites. Detection of pollution was treated as a dichotomous variable such that detection of pollution was coded as one (1) while non-detection was coded as zero (0).

The hotspots for potential cholera-like and vibriosis infections were determined by comparing CMVGI across sites statistically. Since salinity affects the prevalence of *Vibrio* spp. in aquatic milieu, brackish water, and freshwater sampling sites were analyzed independently. To decide on parametric or non-parametric tools for analysis, variables were subjected to Levene test. One-way Analysis of Variance (ANOVA) and Bonferroni Post Hoc Test were employed to compare CMVGI across sites for parametric variables while Welch ANOVA test and Game-Howell Post Hoc test were employed for non-parametric variables.

The specie with the highest CMVGI and any other specie with CMVGI that is not significantly different from that of the species with the highest CMVGI out of the four *Vibrio* spp. were regarded as the *Vibrio* spp. of utmost public health importance in our sampling areas. The different combinations of virulence determinants detected in each of the four *Vibrio* spp. were also noted.

The site having the highest CMVGI served as the reference site for the determination of hotspots among our sampling sites. The site(s) with CMVGI that is not significantly different from the CMVGI of the reference site (the first hotspot) were regarded as additional hotspots for possible cholera and vibriosis outbreaks. The p-value was set at 0.05 for all analyses.

### Electronic supplementary material

Below is the link to the electronic supplementary material.

Supplementary Material 1

## Data Availability

All data generated or analysed during this study are included in this published article [and its supplementary information files].
