# Supplementary material for: Occurrence of virulence determinants in vibrio cholerae, vibrio mimicus, vibrio alginolyticus, and vibrio parahaemolyticus isolates from important water resources of Eastern Cape, South Africa
Source: BMC Microbiol. 2023 Oct 28;23:316. doi: 10.1186/s12866-023-03060-z (PMC10612165; doi:10.1186/s12866-023-03060-z)
Supplement: Supplementary file 1 — Supplementary Material 1 [file 12866_2023_3060_MOESM1_ESM.pdf]

Table S1*:* Detection of virulence determinants in *Vibrio cholerae* from freshwater sample sites

Isolates	sites	Months	ctxA	zot	vpi	toxR	ompU	Tcp	ace	hyla	rtxA	rtxC	ctxB	Total	genotypes	MVGI
320	EL3	$JAN	0	0	0	0	0	0	0	1	0	0	0	1	hylA	0.090909
264	PA6	$JAN	0	0	0	0	0	0	0	1	1	1	0	3	hylA,rtxA,rtxC	0.272727
268	PA6	$JAN	0	0	0	0	0	0	0	1	1	1	0	3	hylA,rtxA,rtxC	0.272727
541	PA6	$FEB	0	0	0	0	0	0	0	1	1	1	0	3	hylA,rtxA,rtxC	0.272727
542	PA6	$FEB	0	0	0	0	0	0	0	1	1	1	0	3	hylA,rtxA,rtxC	0.272727
543	PA6	$FEB	0	0	0	0	0	0	0	1	1	1	0	3	hylA,rtxA,rtxC	0.272727
1240	PA5	$OCT	0	0	0	0	1	0	0	1	1	1	0	4	ompU,hylA,rtxA,rtxC	0.363636
1241	PA5	$OCT	0	0	0	0	1	0	0	1	1	1	0	4	ompU,hylA,rtxA,rtxC	0.363636
157	PA6	$Dec	0	0	0	0	1	0	0	1	1	1	0	4	ompU,hylA,rtxA,rtxC	0.363636
215	PA6	$JAN	0	0	0	0	1	0	0	1	1	1	0	4	ompU,hylA,rtxA,rtxC	0.363636
1243	PA6	$OCT	0	0	0	0	1	0	0	1	1	1	0	4	ompU,hylA,rtxA,rtxC	0.363636
738	PA4	$MAR	0	0	0	0	1	0	0	0	1	1	0	3	ompU,rtxA,rtxC	0.272727
119	PA2	$Dec	0	0	0	1	0	0	0	0	0	0	0	1	toxR	0.090909
123	PA3	$Dec	0	0	0	1	0	0	0	0	0	0	0	1	toxR	0.090909
124	PA3	$Dec	0	0	0	1	0	0	0	0	0	0	0	1	toxR	0.090909
200	PA5	$JAN	0	0	0	1	0	0	0	1	1	1	0	4	toxR,hylA,rtxA,rtxC	0.363636
267	PA5	$JAN	0	0	1	0	0	0	0	1	1	1	0	4	toxR,hylA,rtxA,rtxC	0.363636
534	PA5	$FEB	0	0	0	1	0	0	0	1	1	1	0	4	toxR,hylA,rtxA,rtxC	0.363636
748	PA5	$MAR	0	0	0	1	0	0	0	1	1	1	0	4	toxR,hylA,rtxA,rtxC	0.363636
750	PA5	$MAR	0	0	0	1	0	0	0	1	1	1	0	4	toxR,hylA,rtxA,rtxC	0.363636
896	PA5	$MAY	0	0	0	1	0	0	0	1	1	1	0	4	toxR,hylA,rtxA,rtxC	0.363636
540	PA6	$FEB	0	0	0	1	0	0	0	1	1	1	0	4	toxR,hylA,rtxA,rtxC	0.363636
766	PA6	$MAR	0	0	0	1	0	0	0	1	1	1	0	4	toxR,hylA,rtxA,rtxC	0.363636
661	EL4	$FEB	0	0	0	1	1	0	0	1	1	1	0	5	toxR,ompU,hylA,rtxA,rtxC	0.454545
662	EL4	$FEB	0	0	0	1	1	0	0	1	1	1	0	5	toxR,ompU,hylA,rtxA,rtxC	0.454545
663	EL4	$FEB	0	0	0	1	1	0	0	1	1	1	0	5	toxR,ompU,hylA,rtxA,rtxC	0.454545
509	PA1	$FEB	0	0	0	1	1	0	0	1	1	1	0	5	toxR,ompU,hylA,rtxA,rtxC	0.454545
718	PA3	$MAR	0	0	0	1	1	0	0	1	1	1	0	5	toxR,ompU,hylA,rtxA,rtxC	0.454545
209	PA4	$JAN	0	0	0	1	1	0	0	1	1	1	0	5	toxR,ompU,hylA,rtxA,rtxC	0.454545
736	PA4	$MAR	0	0	0	1	1	0	0	1	1	1	0	5	toxR,ompU,hylA,rtxA,rtxC	0.454545
145	PA5	$Dec	0	0	0	1	1	0	0	1	1	1	0	5	toxR,ompU,hylA,rtxA,rtxC	0.454545
149	PA5	$Dec	0	0	0	1	1	0	0	1	1	1	0	5	toxR,ompU,hylA,rtxA,rtxC	0.454545
152	PA5	$Dec	0	0	0	1	1	0	0	1	1	1	0	5	toxR,ompU,hylA,rtxA,rtxC	0.454545

Isolates	sites	Months	ctxA	zot	vpi	toxR	ompU	Tcp	ace	hyla	rtxA	rtxC	ctxB	Total	genotypes	MVGI
743	PA5	$MAR	0	0	0	1	1	0	0	1	1	1	0	5	toxR,ompU,hylA,rtxA,rtxC	0.454545
745	PA5	$MAR	0	0	0	1	1	0	0	1	1	1	0	5	toxR,ompU,hylA,rtxA,rtxC	0.454545
155	PA6	$Dec	0	0	0	1	1	0	0	1	1	1	0	5	toxR,ompU,hylA,rtxA,rtxC	0.454545
158	PA6	$Dec	0	0	0	1	1	0	0	1	1	1	0	5	toxR,ompU,hylA,rtxA,rtxC	0.454545
202	PA6	$JAN	0	0	0	1	1	0	0	1	1	1	0	5	toxR,ompU,hylA,rtxA,rtxC	0.454545
754	PA6	$MAR	0	0	0	1	1	0	0	1	1	1	0	5	toxR,ompU,hylA,rtxA,rtxC	0.454545
755	PA6	$MAR	0	0	0	1	1	0	0	1	1	1	0	5	toxR,ompU,hylA,rtxA,rtxC	0.454545
757	PA6	$MAR	0	0	0	1	1	0	0	1	1	1	0	5	toxR,ompU,hylA,rtxA,rtxC	0.454545
758	PA6	$MAR	0	0	0	1	1	0	0	1	1	1	0	5	toxR,ompU,hylA,rtxA,rtxC	0.454545
763	PA6	$MAR	0	0	0	1	1	0	0	1	1	1	0	5	toxR,ompU,hylA,rtxA,rtxC	0.454545
764	PA6	$MAR	0	0	0	1	1	0	0	1	1	1	0	5	toxR,ompU,hylA,rtxA,rtxC	0.454545
769	PA6	$MAR	0	0	0	1	1	0	0	1	1	1	0	5	toxR,ompU,hylA,rtxA,rtxC	0.454545
770	PA6	$MAR	0	0	0	1	1	0	0	1	1	1	0	5	toxR,ompU,hylA,rtxA,rtxC	0.454545
1242	PA6	$OCT	0	0	0	1	1	0	0	1	1	1	0	5	toxR,ompU,hylA,rtxA,rtxC	0.454545
1244	PA6	$OCT	0	0	0	1	1	0	0	1	1	1	0	5	toxR,ompU,hylA,rtxA,rtxC	0.454545
1245	PA6	$OCT	0	0	0	1	1	0	0	1	1	1	0	5	toxR,ompU,hylA,rtxA,rtxC	0.454545
1246	PA6	$OCT	0	0	0	1	1	0	0	1	1	1	0	5	toxR,ompU,hylA,rtxA,rtxC	0.454545
537	PA5	$FEB	0	0	0	1	0	0	0	0	1	1	0	3	toxR,rtxA,rtxC	0.272727
538	PA5	$FEB	0	0	0	1	0	0	0	0	1	1	0	3	toxR,rtxA,rtxC	0.272727
715	PA2	$MAR	0	0	1	0	0	0	0	1	1	1	0	4	vpi,hylA,rtxA,rtxC	0.363636
291	PA4	$JAN	0	0	1	0	0	0	0	1	1	1	0	4	vpi,hylA,rtxA,rtxC	0.363636
159	PA6	$Dec	0	0	1	0	1	0	0	1	1	1	0	5	vpi,ompU,hylA,rtxA,rtxC	0.454545
290	PA4	$JAN	0	0	1	0	0	0	0	0	1	1	0	3	vpi,rtxA,rtxC	0.272727
533	PA5	$FEB	0	0	1	0	0	1	0	1	1	1	0	5	vpi,tcp,hylA,rtxA,rtxC	0.454545
536	PA5	$FEB	0	0	1	0	0	1	0	1	1	1	0	5	vpi,tcp,hylA,rtxA,rtxC	0.454545
749	PA5	$MAR	0	0	1	1	0	0	0	1	1	1	0	5	vpi,toxR,hylA,rtxA,rtxC	0.454545
895	PA5	$MAY	0	0	1	1	0	1	0	1	1	1	0	6	vpi,toxR,hylA,rtxA,rtxC	0.545455
156	PA6	$Dec	0	0	1	1	0	0	0	1	1	1	0	5	vpi,toxR,hylA,rtxA,rtxC	0.454545
756	PA6	$MAR	0	0	1	1	0	0	0	1	1	1	0	5	vpi,toxR,hylA,rtxA,rtxC	0.454545
759	PA6	$MAR	0	0	1	1	0	0	0	1	1	1	0	5	vpi,toxR,hylA,rtxA,rtxC	0.454545
762	PA6	$MAR	0	0	1	1	0	0	0	1	1	1	0	5	vpi,toxR,hylA,rtxA,rtxC	0.454545
765	PA6	$MAR	0	0	1	1	0	0	0	1	1	1	0	5	vpi,toxR,hylA,rtxA,rtxC	0.454545
713	PA2	$MAR	0	0	1	1	1	0	0	1	1	1	0	6	vpi,toxR,ompU,hylA,rtxA,rtxC	0.545455
716	PA2	$MAR	0	0	1	1	1	0	0	1	1	1	0	6	vpi,toxR,ompU,hylA,rtxA,rtxC	0.545455

Isolates	sites	Months	ctxA	zot	vpi	toxR	ompU	Tcp	ace	hyla	rtxA	rtxC	ctxB	Total	genotypes	MVGI
530	PA4	$FEB	0	0	1	1	1	0	0	1	1	1	0	6	vpi,toxR,ompU,hylA,rtxA,rtxC	0.545455
201	PA6	$JAN	0	0	1	1	1	0	0	1	1	1	0	6	vpi,toxR,ompU,hylA,rtxA,rtxC	0.545455
144	PA5	$Dec	0	0	1	1	1	1	0	1	1	1	0	7	vpi,toxR,ompU,tcp,hylA,rtxA,rtxC	0.636364
741	PA5	$MAR	0	0	1	1	0	1	0	1	1	1	0	6	vpi,toxR,tcp,hylA,rtxA,rtxC	0.545455
742	PA5	$MAR	0	0	1	1	1	0	0	1	1	1	0	6	vpi,toxR,tcp,hylA,rtxA,rtxC	0.545455
744	PA5	$MAR	0	0	1	1	0	1	0	1	1	1	0	6	vpi,toxR,tcp,hylA,rtxA,rtxC	0.545455
746	PA5	$MAR	0	0	1	1	0	1	0	1	1	1	0	6	vpi,toxR,tcp,hylA,rtxA,rtxC	0.545455
461	ALD2	$JAN	0	0	0	0	0	0	0	0	0	0	0	0	ND	0
462	ALD2	$JAN	0	0	0	0	0	0	0	0	0	0	0	0	ND	0
463	ALD2	$JAN	0	0	0	0	0	0	0	0	0	0	0	0	ND	0
181	PA1	$JAN	0	0	0	0	0	0	0	0	0	0	0	0	ND	0
210	PA4	$JAN	0	0	0	0	0	0	0	0	0	0	0	0	ND	0
213	PA4	$JAN	0	0	0	0	0	0	0	0	0	0	0	0	ND	0
270	PA4	$JAN	0	0	0	0	0	0	0	0	0	0	0	0	ND	0
303	PA6	$JAN	0	0	0	0	0	0	0	0	0	0	0	0	ND	0

1 = present, 0 = absent, total = total number of targeted virulence genes detected, MVGI = multiple virulence gene index, ND = none detected

Table S2: Detection of virulence determinants in *Vibrio cholerae* from brackish water sample sites

Isolates	site	Months	ctxA	zot	vpi	toxR	ompU	Tcp	ace	hyla	rtxA	rtxC	ctxB		Total	Genotypes	MVGI
374	SKR	$JAN	0	0	0	0	0	0	0	1	1	1	0		3	hyla,rtxA,rtxC	0.2727273
544	PA7	$FEB	0	0	0	0	0	0	0	1	1	1	0		3	hylA,rtxA,rtxC	0.2727273
607	SKR	$FEB	0	0	0	0	0	0	0	1	1	1	0		3	hylA,rtxA,rtxC	0.2727273
386	SKR	$JAN	0	0	0	0	1	0	0	1	1	1	0		4	ompU,hylA,rtxA,rtxC	0.3636364
387	SKR	$JAN	0	0	0	0	1	0	0	1	1	1	0		4	ompU,hylA,rtxA,rtxC	0.3636364
771	PA7	$MAR	0	0	0	1	0	0	0	1	1	1	0		4	toxR,hylA,rtxA,rtxC	0.3636364
774	PA7	$MAR	0	0	0	1	0	0	0	1	1	1	0		4	toxR,hylA,rtxA,rtxC	0.3636364
544(NI)	PA7	FEB	0	0	0	1	0	0	0	1	1	1	0		4	toxR,hylA,rtxA,rtxC	0.3636364
608	SKR	$FEB	0	0	0	1	0	0	0	1	1	1	0		4	toxR,hylA,rtxA,rtxC	0.3636364
874	SKR	$MAY	0	0	0	1	0	0	0	1	1	1	0		4	toxR,hylA,rtxA,rtxC	0.3636364
372	SKR	$JAN	0	0	0	1	1	0	0	1	1	1	0		5	toxR,ompU,hylA,rtxA,rtxC	0.4545455
390	SKR	$JAN	0	0	0	1	1	0	0	1	1	1	0		5	toxR,ompU,hylA,rtxA,rtxC	0.4545455
606	SKR	$FEB	0	0	0	1	1	0	0	0	1	1	0		4	toxR,ompU,rtxA,rtxC	0.3636364
98	EL5	$Dec	0	0	0	1	0	0	0	0	1	1	0		3	toxR,rtxA,rtxC	0.2727273
329C	EL5	$JUN	0	0	0	1	0	0	0	0	1	1	0		3	toxR,rtxA,rtxC	0.2727273

Isolates	site	Months	ctxA	zot	vpi	toxR	ompU	Tcp	ace	hyla	rtxA	rtxC	ctxB		Total	Genotypes	MVGI
375	SKR	$JAN	0	0	1	1	1	0	0	1	1	1	0		6	vpi,toxR,ompU,hylA,rtxA,rtxC	0.5454545
377	SKR	$JAN	0	0	1	1	1	0	0	1	1	1	0		6	vpi,toxR,ompU,hylA,rtxA,rtxC	0.5454545
599	SR	$FEB	0	0	1	1	0	1	0	1	1	1	0		6	vpi,toxR,tcp,hylA,rtxA,rtxC	0.5454545
600	SR	$FEB	0	0	1	1	0	1	0	1	1	1	0		6	vpi,toxR,tcp,hylA,rtxA,rtxC	0.5454545
602	SR	$FEB	0	1	1	1	1	0	0	1	1	1	0		7	Zot,vpi,toxR,ompU,hylA,rtxA,rtxC	0.6363636
183C	EL5	$MAY	0	0	0	0	0	0	0	0	0	0	0		0	ND	0
196C	EL5	$MAY	0	0	0	0	0	0	0	0	0	0	0		0	ND	0
209C	EL5	$MAY	0	0	0	0	0	0	0	0	0	0	0		0	ND	0
330C	EL5	$JUN	0	0	0	0	0	0	0	0	0	0	0		0	ND	0
334C	EL5	$JUN	0	0	0	0	0	0	0	0	0	0	0		0	ND	0
373	SKR	$JAN	0	0	0	0	0	0	0	0	0	0	0		0	ND	0
388	SKR	$JAN	0	0	0	0	0	0	0	0	0	0	0		0	ND	0
605	SKR	$FEB	0	0	0	0	0	0	0	0	0	0	0		0	ND	0
230C	SKR	$MAY	0	0	0	0	0	0	0	0	0	0	0		0	ND	0

1 = present, 0 = absent, total = total number of targeted virulence genes detected, MVGI = multiple virulence gene index, ND = none detected

Table S3: Detection of virulence determinants in *Vibrio mimicus* from freshwater sample sites

Isolates	sites	Months	ctxA	zot	vpi	toxR	ompU	Tcp	ace	hyla	rtxA	rtxC	ctxB	Total	Genotypes	MVGI
691	EL2	$FEB	0	0	0	0	1	0	0	0	0	0	0	1	ompU	0.090909
693	EL2	$FEB	0	0	0	0	1	0	0	0	0	0	0	1	ompU	0.090909
292	PA3	$JAN	0	0	0	1	0	0	0	0	0	0	0	1	toxR	0.090909
529	PA4	$FEB	0	0	1	0	0	0	0	1	1	1	0	4	vpi,hylA,rtxA,rtxC	0.363636
739	PA4	$MAR	0	0	0	0	1	0	0	1	1	1	0	4	vpi,hylA,rtxA,rtxC	0.363636
363	EL1	$JAN	0	0	1	0	1	0	0	0	0	0	0	2	vpi,ompU	0.181818
712	PA2	$MAR	0	0	1	0	1	0	0	0	0	0	0	2	vpi,ompU	0.181818
730	PA3	$MAR	0	0	1	0	1	0	0	0	1	1	0	4	vpi,ompU,rtxA,rtxC	0.363636
532	PA5	$FEB	0	0	1	0	1	0	0	0	1	1	0	4	vpi,ompU,rtxA,rtxC	0.363636
535	PA5	$FEB	0	0	1	0	1	0	0	0	1	1	0	4	vpi,ompU,rtxA,rtxC	0.363636
75	EL1	$Dec	0	0	1	1	0	0	0	0	0	0	0	2	vpi,toxR	0.181818
136	PA4	$Dec	0	0	1	1	0	0	0	1	1	1	0	5	vpi,toxR,hylA,rtxA,rtxC	0.454545
288	PA4	$JAN	0	0	1	1	0	0	0	1	1	1	0	5	vpi,toxR,hylA,rtxA,rtxC	0.454545
286	PA2	$JAN	0	0	0	0	0	0	0	0	0	0	0	0	ND	0

1 = present, 0 = absent, total = total number of targeted virulence genes detected, MVGI = multiple virulence gene index, ND = none detected

Table S4: Detection of virulence determinants in *Vibrio mimicus* from brackish water sample sites

Isolates	Sites	Months	ctxA	zot	vpi	toxR	ompU	Tcp	ace	hyla	rtxA	rtxC	ctxB	**Total**	Genotypes
328C	EL5	$JUN	0	0	1	0	0	0	0	0	1	1	0	3	vpi,rtxA,rtxC
101	EL6	$Dec	0	0	1	0	0	0	0	0	1	1	0	3	vpi,rtxA,rtxC
665	EL6	$FEB	0	0	0	1	0	0	0	0	1	1	0	3	toxR,rtxA,rtxC
667	EL6	$FEB	0	0	0	0	0	0	0	0	0	0	0	0	ND
164	PA7	$Dec	0	0	0	0	0	0	0	0	0	0	0	0	ND
165	PA7	$Dec	0	0	0	0	0	0	0	0	0	0	0	0	ND
371	SKR	$JAN	0	0	0	0	0	0	0	0	0	0	0	0	ND
370	SR	$JAN	0	0	0	0	0	0	0	0	0	0	0	0	ND

1 = present, 0 = absent, total = total number of targeted virulence genes detected, MVGI = multiple virulence gene index, ND = none detected

Table S5: Detection of virulence determinants in *Vibrio alginolyticus* from freshwater sample sites

**Isolates**	**Sites**	**Months**	**tlh**	**trh**	**tdh**	**vpc**	**vop**	**vgrg**	**hcp**	**vpi**	**Genotypes**	**MVGI**
520	PA4	$FEB	1	1	0	0	0	0	0	0	trh	0.125
514	PA2	$FEB	0	0	0	0	0	0	0	0	ND	0
517	PA3	$FEB	0	0	0	0	0	0	0	0	ND	0
521	PA4	$FEB	0	0	0	0	0	0	0	0	ND	0
528	PA4	$FEB	0	0	0	0	0	0	0	0	ND	0

Table S6: Detection of virulence determinants in *Vibrio alginolyticus* from brackish sample sites

**Isolates**	**Sites**	**Months**	**tlh**	**trh**	**tdh**	**vpc**	**vop**	**vgrg**	**hcp**	**vpi**	**Total**	**Genotypes**	**MVGI**
100	EL6	$Dec	1	0	0	0	0	1	1	0	3	tlh,vgrg,hcp	0.375
104	EL6	$Dec	1	0	0	0	0	1	1	0	3	tlh,vgrg,hcp	0.375
102	EL6	$Dec	1	0	0	0	0	1	1	1	4	tlh,vgrg,hcp,vpi	0.5
103	EL6	$Dec	1	0	0	0	0	1	1	1	4	tlh,vgrg,hcp,vpi	0.5
168	PA7	$Dec	1	0	0	1	0	1	1	0	3	tlh,vgrg,hcp	0.375
653	EL5	$FEB	1	0	1	1	0	1	1	0	3	tlh,vgrg,hcp	0.375
647	EL6	$FEB	0	0	1	0	0	1	1	0	3	tdh,vgrg,hcp	0.375
666	EL6	$FEB	1	1	0	0	0	1	1	0	4	tlh,trh,vgrg,hcp	0.5
668	EL6	$FEB	1	1	0	0	0	1	1	0	4	tlh,trh,vgrg,hcp	0.5
644	EL6	$FEB	1	0	0	0	0	1	1	1	4	tlh,vgrg,hcp,vpi	0.5

Isolates	Sites	Months	tlh	trh	tdh	vpc	vop	vgrg	hcp	vpi	Total	Genotypes	MVGI
645	EL6	$FEB	1	0	0	0	0	1	1	1	4	tlh,vgrg,hcp,vpi	0.5
646	EL6	$FEB	1	0	0	0	0	1	1	1	4	tlh,vgrg,hcp,vpi	0.5
545	PA7	$FEB	1	0	0	1	0	1	1	0	4	tlh,vpc,vgrg,hcp	0.5
550	PA7	$FEB	0	0	0	0	0	0	0	0			0
578	SKR	$FEB	1	0	0	1	0	1	1	0	4	tlh,vpc,vgrg,hcp	0.5
581	SKR	$FEB	1	0	0	1	0	1	1	0	4	tlh,vpc,vgrg,hcp	0.5
579	SKR	$FEB	1	0	0	1	0	1	1	1	5	tlh,vpc,vgrg,hcp,vpi	0.625
572	SR	$FEB	1	0	0	1	0	1	1	0	4	tlh,vpc,vgrg,hcp	0.5
573	SR	$FEB	1	0	0	1	0	1	1	0	4	tlh,vpc,vgrg,hcp	0.5
575	SR	$FEB	1	0	0	1	0	1	1	0	4	tlh,vpc,vgrg,hcp	0.5
577	SR	$FEB	1	0	0	1	0	1	1	0	4	tlh,vpc,vgrg,hcp	0.5
570	SR	$FEB	1	0	0	1	0	1	1	1	5	tlh,vpc,vgrg,hcp,vpi	0.625
571	SR	$FEB	1	0	0	1	0	1	1	1	5	tlh,vpc,vgrg,hcp,vpi	0.625
576	SR	$FEB	1	0	0	1	0	1	1	1	5	tlh,vpc,vgrg,hcp,vpi	0.625
332	EL5	$JAN	1	0	0	0	0	1	1	0	3	tlh,vgrg,hcp	0.375
335	EL5	$JAN	1	0	0	0	0	1	1	0	3	tlh,vgrg,hcp	0.375
402a	SKR	$JAN	1	0	0	1	0	0	0	0	2	tlh,vpc	0.25
378	SKR	$JAN	1	0	0	1	0	1	1	0	4	tlh,vpc,vgrg,hcp	0.5
380	SKR	$JAN	1	0	0	1	0	1	1	0	4	tlh,vpc,vgrg,hcp	0.5
393	SKR	$JAN	0	0	0	1	0	1	1	0	3	vpc,vgrg,hcp	0.375
400	SKR	$JAN	0	0	0	1	0	1	1	0	3	vpc,vgrg,hcp	0.375
366	SR	$JAN	1	0	0	1	0	1	1	0	4	tlh,vpc,vgrg,hcp	0.5
369	SR	$JAN	1	0	0	1	0	1	1	0	4	tlh,vpc,vgrg,hcp	0.5
404	SR	$JAN	1	0	0	1	0	1	1	0	4	tlh,vpc,vgrg,hcp	0.5
367	SR	$JAN	1	0	0	1	0	1	1	1	5	tlh,vpc,vgrg,hcp,vpi	0.625
365	SR	$JAN	1	0	0	1	0	1	1	1	5	tlh,vpc,vgrg,hcp,vpi	0.625
402	SR	$JAN	1	0	0	1	0	1	1	1	5	tlh,vpc,vgrg,hcp,vpi	0.625
403	SR	$JAN	1	0	0	1	0	1	1	1	5	tlh,vpc,vgrg,hcp,vpi	0.625
405	SR	$JAN	1	0	0	1	0	1	1	1	5	tlh,vpc,vgrg,hcp,vpi	0.625
406	SR	$JAN	1	0	0	1	0	1	1	1	5	tlh,vpc,vgrg,hcp,vpi	0.625
407	SR	$JAN	1	0	0	1	0	1	1	1	5	tlh,vpc,vgrg,hcp,vpi	0.625
948	EL5	$MAY	1	0	1	1	0	1	1	0	5	tlh,tdh,vpc,vgrg,hcp	0.625
951	EL5	$MAY	1	0	1	1	0	1	1	0	5	tlh,tdh,vpc,vgrg,hcp	0.625
955	EL5	$MAY	1	0	1	1	0	1	1	0	5	tlh,tdh,vpc,vgrg,hcp	0.625

Isolates	Sites	Months	tlh	trh	tdh	vpc	vop	vgrg	hcp	vpi	Total	Genotypes	MVGI
1003	EL5	$MAY	1	0	1	1	0	1	1	0	5	tlh,tdh,vpc,vgrg,hcp	0.625
208C	EL5	$MAY	0	0	0	0	0	0	0	0			0
994	EL6	$MAY	1	1	1	0	0	1	1	0	5	tlh,trh,tdh,vgrg,hcp	0.625
959	EL6	$MAY	1	0	0	0	0	1	1	0	3	tlh,vgrg,hcp	0.375
964	EL6	$MAY	1	0	0	0	0	1	1	0	3	tlh,vgrg,hcp	0.375
965	EL6	$MAY	1	0	0	0	0	1	1	0	3	tlh,vgrg,hcp	0.375
966	EL6	$MAY	1	0	0	0	0	1	1	0	3	tlh,vgrg,hcp	0.375
995	EL6	$MAY	1	0	0	0	0	1	1	0	3	tlh,vgrg,hcp	0.375
960	EL6	$MAY	0	0	0	0	0	1	1	0	2	vgrg,hcp	0.25
963	EL6	$MAY	0	0	0	0	0	1	1	0	2	vgrg,hcp	0.25
189C	SKR	$MAY	1	1	1	0	0	0	0	0	3	tlh,tdh,trh	0.375
155C	SKR	$MAY	1	0	1	1	0	1	1	0	5	tlh,tdh,vpc,vgrg,hcp	0.625
188C	SKR	$MAY	1	0	0	1	0	1	1	0	4	tlh,vpc,vgrg,hcp	0.5
1258	SKR	$NOV	1	0	0	1	0	1	1	0	4	tlh,vpc,vgrg,hcp	0.5
1260	SKR	$NOV	1	0	0	1	0	1	1	0	4	tlh,vpc,vgrg,hcp	0.5
1251	SR	$NOV	1	0	0	1	0	1	1	0	4	tlh,vpc,vgrg,hcp	0.5

Table S7 Detection of virulence determinants in *Vibrio parahaemolyticus* from freshwater sample sites

Isolates	site	Months	tlh	trh	tdh	vpc	vop	vgrg	hcp	vpi	Total	Genotype	MVGI
1028	ALD2	$JUN	0	0	1	0	0	0	0	0	1	tdh	0.125

Table S8 Detection of virulence determinants in *Vibrio parahaemolyticus* from brackish water sample sites

Isolates	site	Months	tlh	trh	tdh	vpc	vop	vgrg	hcp	vpi	Total	Genotypes	MVGI
1261	SKR	$NOV	1	0	0	0	0	0	0	0	1	tlh	0.125
1278	SKR	$NOV	1	0	0	0	0	0	0	0	1	tlh	0.125
338	EL5	$JAN	1	0	0	0	0	1	1	0	3	tlh,vgrg,hcp	0.375
643	EL6	$FEB	1	0	0	0	0	1	1	1	4	tlh,vgrg,hcp,vpi	0.5
648	EL6	$FEB	1	0	0	0	0	1	1	1	4	tlh,vgrg,hcp,vpi	0.5
546	PA7	$FEB	1	0	0	0	0	1	1	1	4	tlh,vgrg,hcp,vpi	0.5
215C	SKR	$MAY	1	0	0	1	0	1	1	0	4	tlh,vpc,vgrg,hcp	0.5
568	SR	$FEB	1	0	0	1	0	1	1	0	4	tlh,vpc,vgrg,hcp	0.5

574	SR	$FEB	1	0	0	1	0	1	1	0	4	tlh,vpc,vgrg,hcp	0.5
1250	SR	$NOV	1	0	0	1	0	1	1	0	4	tlh,vpc,vgrg,hcp	0.5
1252	SR	$NOV	1	0	0	1	0	1	1	0	4	tlh,vpc,vgrg,hcp	0.5
185C	SKR	$MAY	1	0	0	1	0	1	1	1	5	tlh,vpc,vgrg,hcp,vpi	0.625
383	SKR	$JAN	1	0	0	1	0	0	0	1	3	tlh,vpc,vpi	0.375
580	SKR	$FEB	1	0	0	1	0	0	0	1	3	tlh,vpc,vpi	0.375
583	SKR	$FEB	1	0	0	1	0	0	0	1	3	tlh,vpc,vpi	0.375
260	PA7	$JAN	0	0	0	0	1	1	1	0	3	vop,vgrg,hcp	0.375

GEL PICTURES

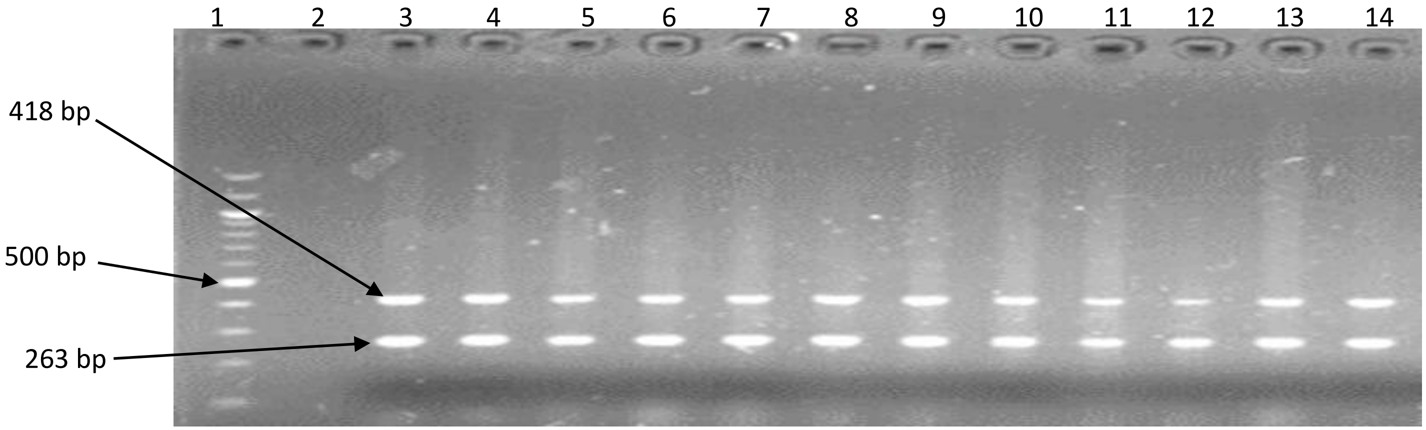

Figure S1: Gel pictures sample showing PCR duplex amplification products of the specific regions of *rtx* A and *rtx* C genes Lane 1 = 100bp molecular marker, lane 2 = negative control, lane 3 = positive control and lanes 4 -14 = positive isolates.

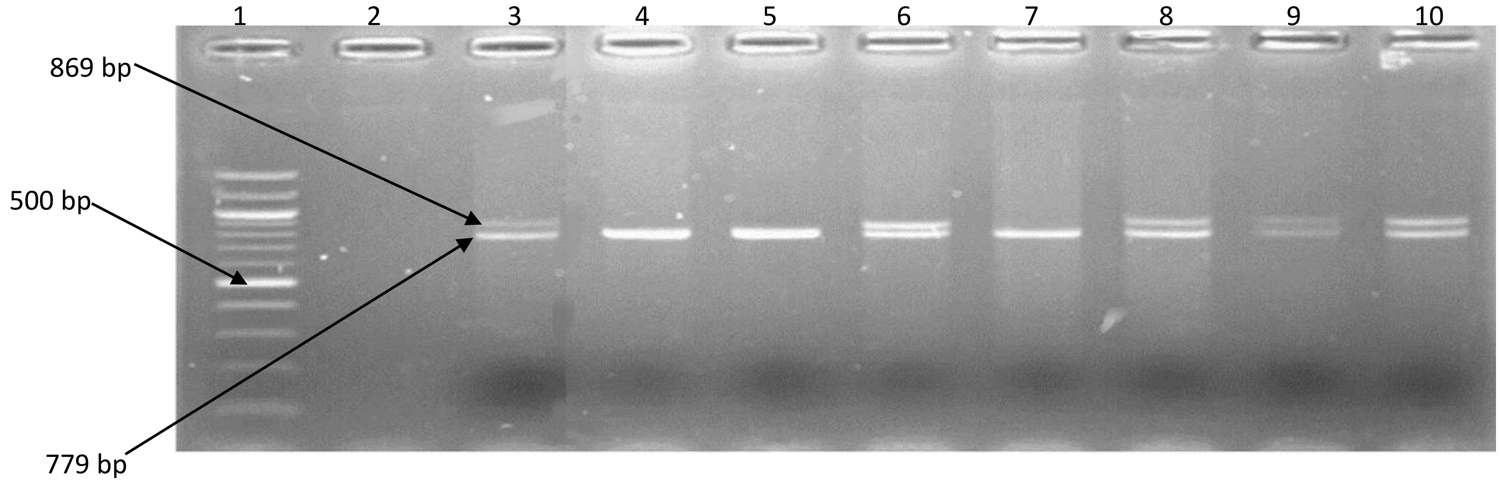

Figure S2: Gel pictures sample showing PCR duplex amplification products of the specific regions of *tox*R and *omp*U genes Lane 1 = 100bp molecular marker, lane 2 = negative control, lane 3 = positive control and lanes 4 -10 = ToxR positive isolates, lanes 6, 8-10 = OmpU positive isolates.

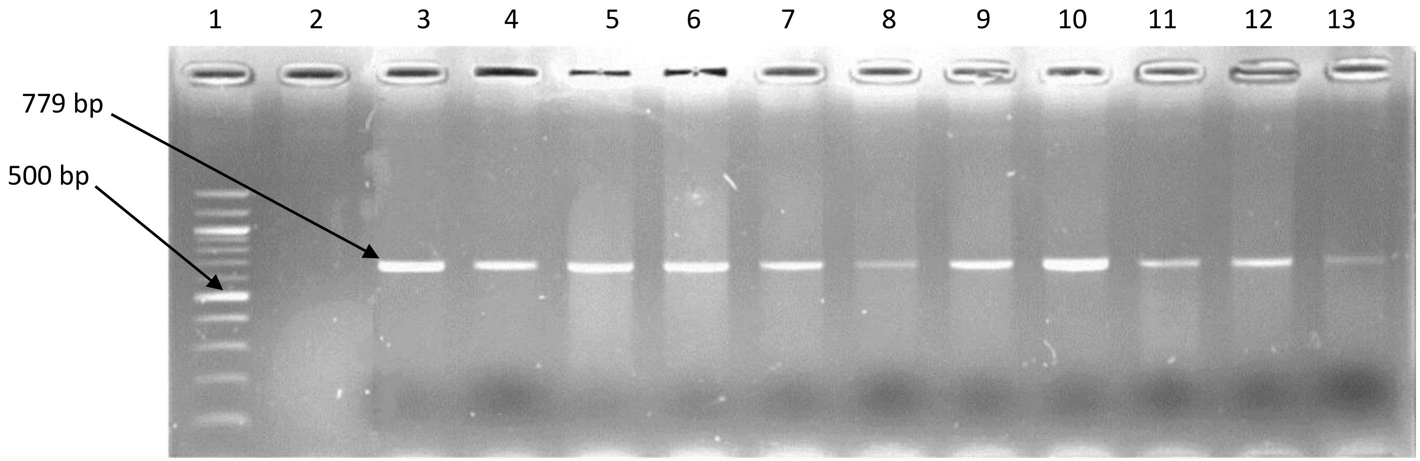

Figure S3: Gel pictures sample showing PCR sigleplex amplification product of the specific region of *vpi* gene Lane 1 = 100bp molecular marker, lane 2 = negative control, lane 3 = positive control and lanes 4 -13 = positive isolates.

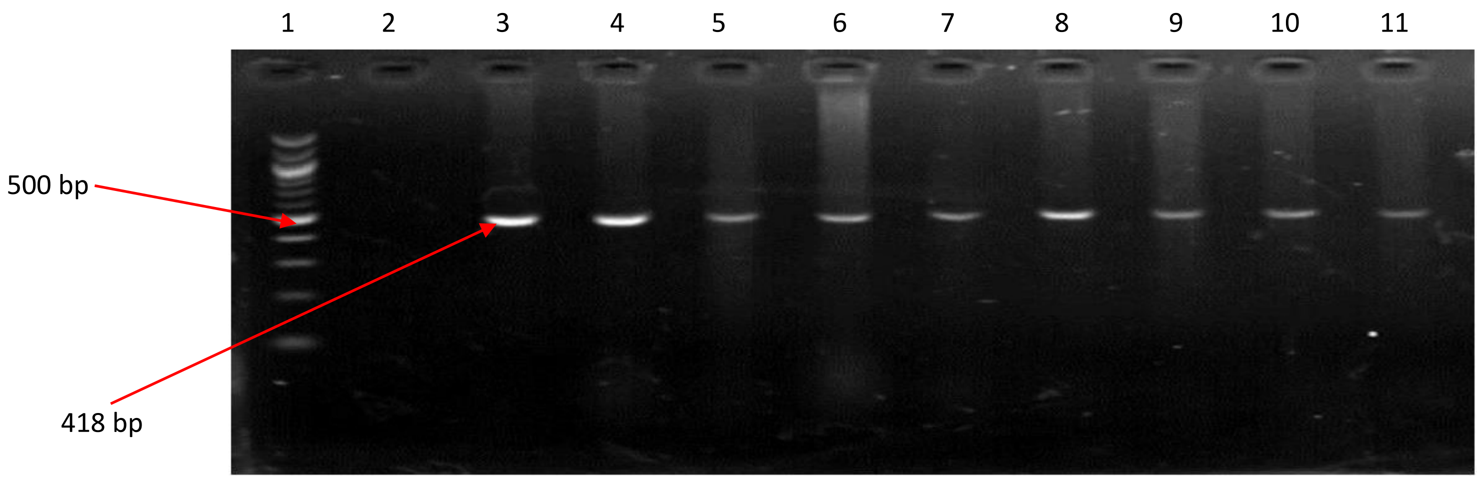

Figure S4: Gel pictures sample showing PCR sigleplex amplification product of the specific region of *hylA* gene Lane 1 = 100bp molecular marker, lane 2 = negative control, lane 3 = positive control and lanes 4 -13 = positive isolates.

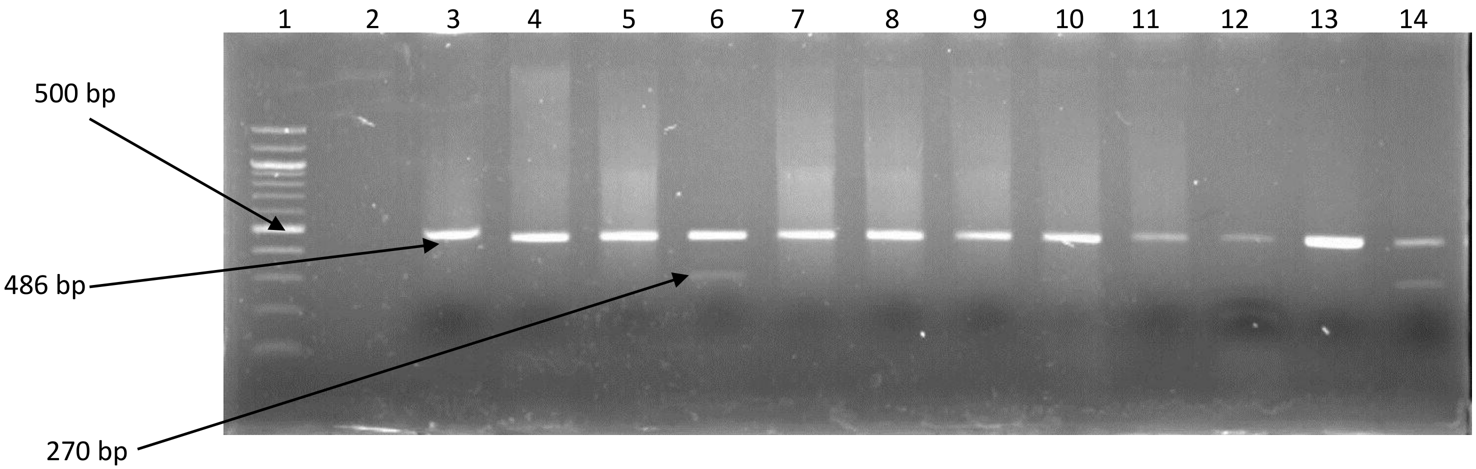

Figure S5: Gel pictures sample showing PCR duplex amplification products of the specific regions of *tdh* and *trh* genes Lane 1 = 100bp molecular marker, lane 2 = negative control, lane 3 -14 = TDH positive isolates, Lanes 6&14 = TRH positive isolates.

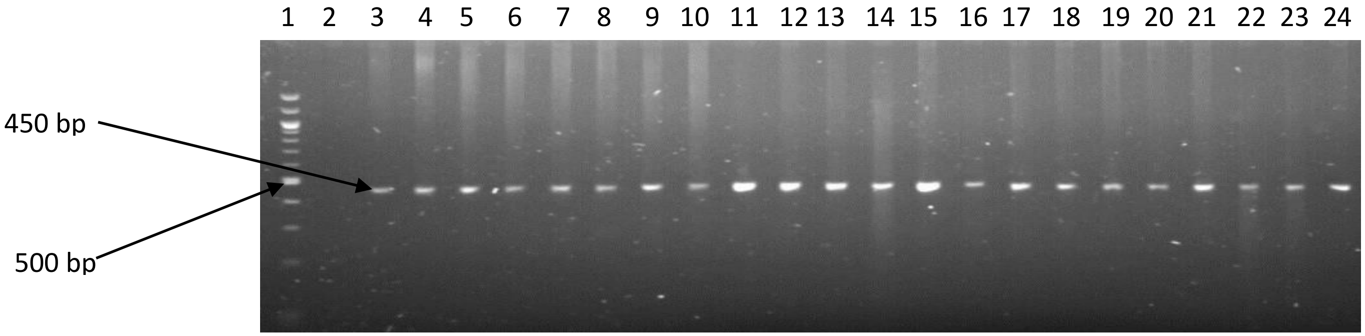

Figure S6: Gel pictures sample showing PCR single[lex amplification product of the specific region of *tlh* gene Lane 1 = 100bp molecular marker, lane 2 = negative control, lane 3 = positive control, lane 4 - 24 = *tlh* positive isolates.

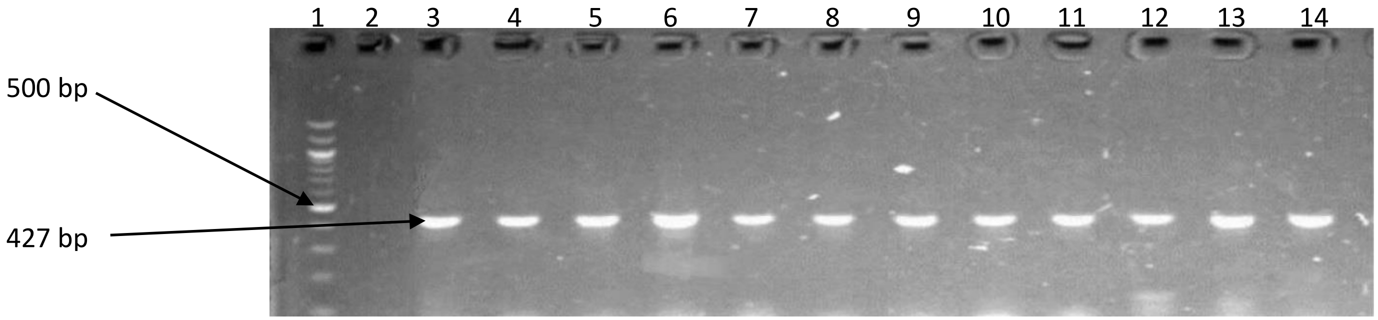

Figure S7: Gel pictures sample showing PCR single[lex amplification product of the specific region of *vppC* gene Lane 1 = 100bp molecular marker, lane 2 = negative control, lane 3 = positive control, lane 4 - 24 = *tlh* positive isolates.

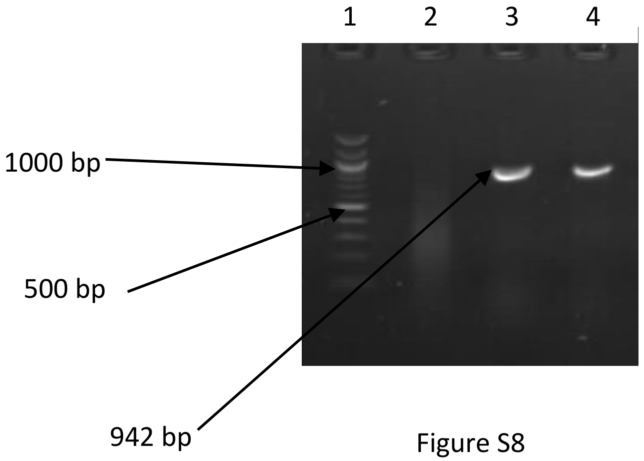

Figure S8

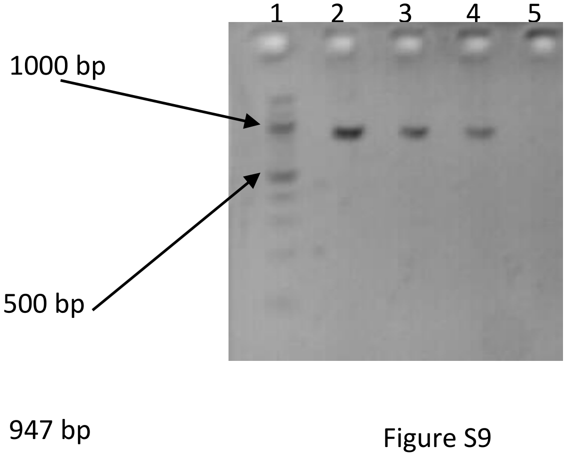

Figure S9

Figure S8: Gel pictures sample showing PCR singleplex amplification product of the specific region of *vopB2* gene Lane 1 = 100bp molecular marker, lane 2 = negative control, lane 3 = positive control, lane 4 = *vopB2* positive isolates.

Figure S9: Gel pictures sample showing PCR singleplex amplification product of the specific region of *zot* gene Lane 1 = 100bp molecular marker, lane 2 = positive control, lane 3&4 = zot positive isolate, lane 5 = negative control. Note: Lane 3&4 contain amplicon for the only *V. cholerae* that [illegible]

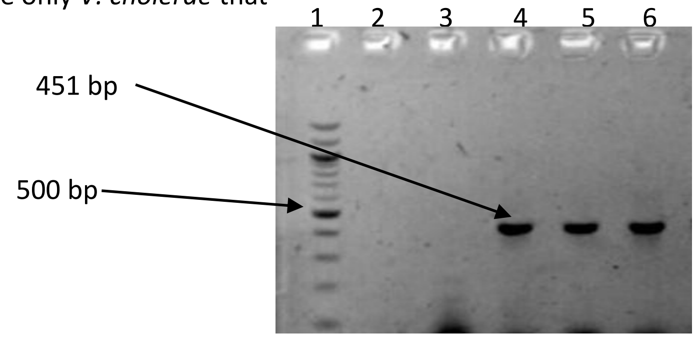

Figure S10: Gel pictures sample showing PCR singleplex amplification product of the specific region of TCP gene Lane 1 = 100bp molecular marker, lane 2 = negative control, lane 3 = isolate that is negative for *tcp* gene, lane 4 – 6 = TCP positive isolates

Table S9: List of primers and PCR conditions

Confirmation of *V. cholerae* isolates as O1, O139 non-O1/non-O139 serogroup										
					35 cycles					
Genes	Gene Products	Primer's sequence	AMS (bp)	ID(°C)-min	DE(°C) -min	AN(°C)-min	EX(°C) -min	FE(°C) – min	PCR types	Ref
O1-rfb	O1 specific LPS	F:5'-GTTTCACTGAACAGATGGG-3'<br>R:5'-GGTCATCTGTAAGTACAAC-3'	192	94-5	94-1	55-1	72-1	72-7	Duplex	Hoshino et al., 1998
O139-rfb	O139 specific LPS	F:5'-AGCCTCTTTATTACGGGTGG-3'<br>R:5'-GTCAAACCCGATCGTAAAGG-3'	449							
Detection of virulence determinants										
					35 cycles					
Gene	Gene Products	Primer's sequence	AMS (bp)	ID(°C)-min	DE(°C)-min	AN(°C)-min	EX(°C)-min	FE(°C)-min	PCR types	Ref
rtxA	repeat like toxin subunit A	F:5′-CTG AAT ATG AGT GGG TGA CTT ACG-3′<br>R:5′GTG TAT TGT TCG ATA TCC GCT ACG-3′	418	94-2	94-1	55-1	72-1	72-7	Duplex	Chow et al 2001
rtxC	repeat like toxin subunit C	F:5′-CGA CGA AGA TCA TTG ACG AC-3′<br>R:5′-CAT CGT CGT TAT GTG GTT GC-3′	263							
toxR	transmembrane transcriptional activator	F:5′-CCTTCGATCCCCTAAGCAATAC-3′<br>R:5′-AGGGTTAGCAACGATGCGTAAG-3′	779	94-2	94-1	60-1	72-1	72-7	singleplex	Rivera 2001
ompU	pore-forming proteins of the outer membrane	F:5′-ACGCTGACGGAATCAACCAAAG-3′<br>R:5′-GCGGAAGTTTGGCTTGAAGTAG-3′	869	94-2	94-1	60-1	72-1	72-7	singleplex	Rivera 2001
ctxA	cholera toxin subunit A	F:5′-CTC AGA CGG GAT TGT TAG GCA CG-3′<br>R:5′-TCT ATC TCT GTA GCC CCT ATT ACG-3′	301	94-3	94-1	60-1.5	72-1.5	72-7	singleplex	Shirai et al 1991
ctxB	repeat like toxin subunit B	F:5′-GAT ACA CAT AAT AGA ATT AAG GAT G-3′<br>R:5′-GGT TGC TTC TCA TCA TCG AAC CAC-3′	461	95-4	95-1	55-1	72-1	72-7	singleplex	Olsvik1993
vpi	vibrio pathogenicity iceland	F:5′-GCA ATT TAG GGG CGC GAC GT-3′<br>R:5′-CCG CTC TTT CTT GAT CTG GTA G-3′	680	94-2	94-1	52-1	72-1	72-7	singleplex	Sechi et al 2000
hylA El Tor	haemolysin	F: 5′-GAGCCGGCATTCATCTGAAT-3′<br>R: 5′-CTCAGCGGGCTAATACGGTTTA-3′	481	94-2	94-1	60-1	72-1	72-7	singleplex	Rivera 2001
Genes Gene Products		Primer's sequence	AMS (bp)	ID(°C)-min	35 cycles DE(°C) -min	AN(°C)-min	EX(°C) -min	FE(°C) – min	PCR types	Ref
tcpA El Tor	Toxin co-regulated pili	F: 5′-CAC GAT AAG AAA ACC GGT CAA GAG-3′<br>R: 5′-CGA AAG CAC CTT CTT TCA CGT TG-3′	451	94-2	94-1	60-1	72-1	72-7	singleplex	Rivera 2001
Zot	zonula occludens toxin	F: 5′-TCGCTTAACGATGGCGCGTTTT-3′<br>R: 5′- AACCCCGTTTCACTTCTACCCA-3′	947	94-10	94-1	59-1	72-2	72-10	Singleplex	Menezes *et al* ., 2014
tdh	thermostable direct hemolysin	F: 5′-GTA AAG GTC TCT GAC TTT TGG AC-3′<br>R: 5′-TGG AAT AGA ACC TTC ATC TTC ACC-3′	270	93 °C-5	92-1	55-1	72-1.5	75-7	Duplex	Bhattacharyya et al 2012)

trh	tdh-related hemolysin	F: 5’-TTG GCT TCG ATA TTT TCA GTA TCT-3’<br>R: 5’-CAT AAC AAA CAT ATG CCC ATT TCC G-3’	486							
tlh	thermolabile hemolysin	F: 5’-AAAGCGGATTATGCAGAAGCACTG-3’<br>R: 5’-GCTACTTTCTAGCATTTTCTCTGC-3’	450	93 °C-5	92-1	55-1	72-1.5	75-7	Singleplex	Bhattacharyya et al 2012)
vppC	metalloprotease gene	F: 5’-GTCGGATTCAGAGTCGGTATTTAG-3’<br>R: 5’-GTTCCACTGCCCACCAAACA-3’	427	93-15	92-1	53-1	72-1.5	75-7	Singleplex	(Hernández-Robles et al., 2016b)
vopB2	translocation proteins	F: 5’-F:GAGCCTGTTGCTCTATGGAGCCAGG-3’<br>R: 5’-CGACACAGAACGCAATGCTTGCTCG-3’	942	93-15	92-1	52-1	72-1.5	75-7	Singleplex	(Jones et al., 2012)
vgrG	valine-glycine repeat protein G	F: 5’-GAAGACGAAGCGAACCAAG-3’<br>R: 5’-ATTGAACCATCACTGTTCATCAC-3’	533	93-15	92-1	54-0.5	72-1.5	75-7	duplex	(Hernández-Robles et al., 2016b)
hcp	Hemolysin-coregulated protein	F: 5’-CAAGAAGCTCACGTTGATG-3’<br>R: 5’-TTGATTGAGTAGTAGTGCTCTTG-3’	248							
ace	accessory cholera enterotoxin	F:5’-TAAGGATGTGCTTATGATGGACACCC-3’<br>R:5’-CGTGATGAATAAAGATACTCATAGG-3’	316	94-2	94-1	62-1	72-1	72-10	singleplex	(Singh et al., 2002)

Key: AMS=amplicon size, ID=initial denaturation, DE=denaturation, AN=annealing, EX=extension, FE=final extension, Ref=references

Table S10: Post Hoc analysis: The comparison of isolates and sampling sites CMVGI

Freshwater Isolates

(I) Sites			Mean Difference	Std. Error	Sig.	95% Confidence Lower	Upper
Bonferroni	VC	VM	.10485	.04573	.072	-.0065	.2162
		VA	.33309*	.07285	.000	.1557	.5105
	VM	VC	-.10485	.04573	.072	-.2162	.0065
		VA	.22825*	.08239	.020	.0276	.4289
	VA	VC	-.33309*	.07285	.000	-.5105	-.1557
		VM	-.22825*	.08239	.020	-.4289	-.0276

brackish water isolates

(I) Sites			Mean Difference	Std. Error	Sig.	95% Confidence Lower	Upper
Games-Howell	VC	VM	.17359	.06330	.061	-.0068	.3540
		VA	-.19914*	.04291	.000	-.3141	-.0842
		VP	-.14601*	.05169	.035	-.2843	-.0077
	VM	VC	-.17359	.06330	.061	-.3540	.0068
		VA	-.37273*	.05306	.000	-.5386	-.2068
		VP	-.31960*	.06038	.001	-.4957	-.1435
	VA	VC	.19914*	.04291	.000	.0842	.3141
		VM	.37273*	.05306	.000	.2068	.5386
		VP	.05313	.03848	.523	-.0530	.1592
	VP	VC	.14601*	.05169	.035	.0077	.2843
		VM	.31960*	.06038	.001	.1435	.4957
		VA	-.05313	.03848	.523	-.1592	.0530

Freshwater sampling sites

(I) Sites			Mean Difference	Std. Error	Sig.	95% Confidence Lower	Upper
Games-Howell	PA1	PA2	-.01948	.24455	1.000	-4.4795	4.4405
		PA3	.04545	.23909	1.000	-5.1506	5.2415
		PA4	-.01918	.23313	1.000	-6.3175	6.2791
		PA5	-.17832	.22812	.980	-7.7941	7.4375
		PA6	-.17493	.22796	.981	-7.8410	7.4911
		EL1	.04545	.22727	1.000	-7.8406	7.9315
		EL2	.13636	.22727	.994	-7.7497	8.0224
		EL4	-.22727	.22727	.945	-8.1133	7.6588
		ALD2	.19602	.22941	.970	-7.0406	7.4326
	PA2	PA1	.01948	.24455	1.000	-4.4405	4.4795
		PA3	.06494	.11688	1.000	-.3895	.5193
		PA4	.00030	.10416	1.000	-.4104	.4110
		PA5	-.15884	.09241	.761	-.5695	.2518
		PA6	-.15545	.09201	.775	-.5667	.2558
		EL1	.06494	.09029	.998	-.3496	.4795
		EL2	.15584	.09029	.757	-.2587	.5704
		EL4	-.20779	.09029	.487	-.6223	.2067
		ALD2	.21550	.09554	.496	-.1949	.6259
	PA3	PA1	-.04545	.23909	1.000	-5.2415	5.1506
		PA2	-.06494	.11688	1.000	-.5193	.3895
		PA4	-.06463	.09059	.999	-.4211	.2919
		PA5	-.22378	.07679	.276	-.5828	.1352
		PA6	-.22039	.07631	.286	-.5806	.1399
		EL1	0.00000	.07423	1.000	-.3671	.3671
		EL2	.09091	.07423	.937	-.2762	.4580
		EL4	-.27273	.07423	.147	-.6399	.0944
		ALD2	.15057	.08054	.688	-.2072	.5084
		PA1	.01918	.23313	1.000	-6.2791	6.3175

PA4	PA2	-.00030	.10416	1.000	-.4110	.4104
	PA3	.06463	.09059	.999	-.2919	.4211
	PA5	-.15915	.05553	.182	-.3565	.0382
	PA6	-.15575	.05486	.193	-.3517	.0402
	EL1	.06463	.05193	.952	-.1262	.2555
	EL2	.15554	.05193	.161	-.0353	.3464
	EL4	-.20810*	.05193	.027	-.3990	-.0172
	ALD2	.21520	.06061	.057	-.0040	.4344
PA5	PA1	.17832	.22812	.980	-7.4375	7.7941
	PA2	.15884	.09241	.761	-.2518	.5695
	PA3	.22378	.07679	.276	-.1352	.5828
	PA4	.15915	.05553	.182	-.0382	.3565
	PA6	.00339	.02647	1.000	-.0839	.0907
	EL1	.22378*	.01968	.000	.1556	.2919
	EL2	.31469*	.01968	.000	.2465	.3828
	EL4	-.04895	.01968	.321	-.1171	.0192
	ALD2	.37434*	.03693	.001	.2019	.5467
PA6	PA1	.17493	.22796	.981	-7.4911	7.8410
	PA2	.15545	.09201	.775	-.2558	.5667
	PA3	.22039	.07631	.286	-.1399	.5806
	PA4	.15575	.05486	.193	-.0402	.3517
	PA5	-.00339	.02647	1.000	-.0907	.0839
	EL1	.22039*	.01771	.000	.1603	.2805
	EL2	.31129*	.01771	.000	.2512	.3714
	EL4	-.05234	.01771	.131	-.1125	.0078
	ALD2	.37095*	.03592	.002	.1961	.5458
EL1	PA1	-.04545	.22727	1.000	-7.9315	7.8406
	PA2	-.06494	.09029	.998	-.4795	.3496
	PA3	0.00000	.07423	1.000	-.3671	.3671
	PA4	-.06463	.05193	.952	-.2555	.1262
	PA5	-.22378*	.01968	.000	-.2919	-.1556
	PA6	-.22039*	.01771	.000	-.2805	-.1603
	EL2	.09091	0.00000		.0909	.0909
	EL4	-.27273	0.00000		-.2727	-.2727
	ALD2	.15057	.03125	.119	-.0585	.3597
EL2	PA1	-.13636	.22727	.994	-8.0224	7.7497
	PA2	-.15584	.09029	.757	-.5704	.2587
	PA3	-.09091	.07423	.937	-.4580	.2762
	PA4	-.15554	.05193	.161	-.3464	.0353
	PA5	-.31469*	.01968	.000	-.3828	-.2465
	PA6	-.31129*	.01771	.000	-.3714	-.2512
	EL1	-.09091	0.00000		-.0909	-.0909
	EL4	-.36364	0.00000		-.3636	-.3636
	ALD2	.05966	.03125	.682	-.1494	.2687
EL4	PA1	.22727	.22727	.945	-7.6588	8.1133
	PA2	.20779	.09029	.487	-.2067	.6223
	PA3	.27273	.07423	.147	-.0944	.6399
	PA4	.20810*	.05193	.027	.0172	.3990
	PA5	.04895	.01968	.321	-.0192	.1171
	PA6	.05234	.01771	.131	-.0078	.1125
	EL1	.27273	0.00000		.2727	.2727
	EL2	.36364	0.00000		.3636	.3636
	ALD2	.42330*	.03125	.007	.2142	.6324
ALD2	PA1	-.19602	.22941	.970	-7.4326	7.0406
	PA2	-.21550	.09554	.496	-.6259	.1949
	PA3	-.15057	.08054	.688	-.5084	.2072
	PA4	-.21520	.06061	.057	-.4344	.0040
	PA5	-.37434*	.03693	.001	-.5467	-.2019
	PA6	-.37095*	.03592	.002	-.5458	-.1961

	EL1	-.15057	.03125	.119	-.3597	.0585
	EL2	-.05966	.03125	.682	-.2687	.1494
	EL4	-.42330*	.03125	.007	-.6324	-.2142

Brackish water sampling sites

(I) Sites			Mean Difference	Std. Error	Sig.	95% Confidence	
						Lower	Upper
Games-Howell	PA7	EL5	-.00036	.08358	1.000	-.2458	.2451
		EL6	-.11566	.06426	.410	-.3147	.0834
		SR	-.25453*	.06306	.009	-.4515	-.0575
		SKR	-.07774	.06601	.764	-.2795	.1241
	EL5	PA7	.00036	.08358	1.000	-.2451	.2458
		EL6	-.11529	.06610	.429	-.3109	.0803
		SR	-.25416*	.06493	.006	-.4473	-.0611
		SKR	-.07737	.06781	.783	-.2765	.1217
	EL6	PA7	.11566	.06426	.410	-.0834	.3147
		EL5	.11529	.06610	.429	-.0803	.3109
		SR	-.13887*	.03688	.004	-.2436	-.0341
		SKR	.03792	.04174	.892	-.0797	.1555
	SR	PA7	.25453*	.06306	.009	.0575	.4515
		EL5	.25416*	.06493	.006	.0611	.4473
		EL6	.13887*	.03688	.004	.0341	.2436
		SKR	.17679*	.03986	.000	.0647	.2889
	SKR	PA7	.07774	.06601	.764	-.1241	.2795
		EL5	.07737	.06781	.783	-.1217	.2765
		EL6	-.03792	.04174	.892	-.1555	.0797
		SR	-.17679*	.03986	.000	-.2889	-.0647
